# Nanodrug‐Engineered Exosomes Achieve a Jointly Dual‐Pathway Inhibition on Cuproptosis

**DOI:** 10.1002/advs.202413408

**Published:** 2024-12-05

**Authors:** Hanxiao Sun, Yang Zou, Zhengtai Chen, Yan He, Kai Ye, Huan Liu, Lihong Qiu, Yufan Zhang, Yuexue Mai, Xinghong Chen, Zhengwei Mao, Wei Wang, Chenggang Yi

**Affiliations:** ^1^ The Second Affiliated Hospital of Zhejiang University College of Medicine Hangzhou 310000 China; ^2^ College of Chemical and Biological Engineering Zhejiang University Hangzhou Zhejiang 310027 China; ^3^ MOE Key Laboratory of Macromolecular Synthesis and Functionalization Department of Polymer Science and Engineering Zhejiang University Hangzhou Zhejiang 310027 China

**Keywords:** cuproptosis, engineered exosomes, FDX1, wound healing

## Abstract

Cuproptosis, caused by an intracellular overload of copper (Cu) ions and overexpression of ferredoxin 1 (FDX1), is identified for its regulatory role in the skin wound healing process. This study verifies the presence of cuproptosis in skin wound beds and reactive oxygen species‐induced cells model. To address the two pathways leading to cell cuproptosis, a nanodrug‐engineered exosomes is proposed. A Cu‐chelator (Clioquinol, CQ) polydopamine (PDA)‐modified stem cell exosome loaded with siRNA‐FDX1, named EXO^siFDX1‐PDA@CQ^, is designed to efficiently inhibit the two cuproptosis pathways. The functionalized exosomes are loaded into an injectable hydrogel and applied to treat diabetic wounds in mice and acute skin wounds in pigs. The local and controlled release of EXO^siFDX1‐PDA@CQ^ ensures the retention of the therapeutic agent at wound beds, effectively promoting wound healing. The strategy of engineered exosomes with functional nanoparticles (NPs) proposed in this study offers an efficient and scalable new approach for regulating cuproptosis.

## Graphical abstract

The EXO^siFDX1‐PDA@CQ^ designed in this study can release CQ (Clioquinol) to chelate Cu in cells, resulting in the immediate inhibition of cuproptosis. Subsequently, internal siRNA is released to interfere with mRNA transcription and translation, preventing cuproptosis from occurring in the long term by downregulating FDX1 expression.

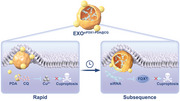

## 1 Introduction

Reactive oxygen species (ROS) are crucial for controlling various processes during typical wound healing,^[^ ^1^ ^]^ such as inflammation,^[^ ^2^ ^]^ cell growth,^[^ ^3^ ^]^ intracellular metal ion levels.^[^ ^4^ ^]^ ROS levels increase sharply after an injury or in a long‐term refractory wound bed,^[^ ^5^ ^]^ causing cells to suffer from excessive oxidative stress injury, which exceedingly activates superoxide dismutase (SOD)^[^ ^6^ ^]^ and Uncontrolable increases copper (Cu) content in cells. As a result, an excessive increase of Cu causes an imbalance in intracellular Cu homeostasis, leading to cuproptosis.^[^ ^7^ ^]^ Cuproptosis is primarily regulated by two pathways: i) intracellular Cu^2+^ overload is reduced to more toxic Cu^+^, which destroys the stability of Fe─S cluster proteins and causes cell death^[^ ^8^ ^]^; ii) the key regulatory molecule ferritin 1 gene (ferredoxin 1 (FDX1) affects the mitochondrial tricarboxylic acid cycle and leads to cell death.^[^ ^9^ ^]^ However, cuproptosis has been reported in various diseases^[^ ^10^ ^]^ but there is a lack of experimental exploration in chronic noninfectious wounds. Therefore, we hypothesized that an intervention strategy that can decrease the Cu content in cells and downregulate the expression of combined current and coming dual spatiotemporal approaches to effectively FDX1 would most efficiently inhibit cuproptosis and promote the skin wound healing process.

Exosomes have recently been extensively used as gene carriers owing to their unique lipid bilayer structure, excellent biocompatibility, and ease of accessibility.^[^ ^11^ ^]^ These vesicles have demonstrated promising therapeutic outcomes in numerous animal disease models and clinical studies.^[^ ^12^ ^]^ However, due to the different loading efficiencies and molecular stabilities of miRNA,^[^ ^13^ ^]^ siRNA^[^ ^14^ ^],^ and drugs^[^ ^15^ ^]^ in exosomes, exosomes are currently primarily used as single‐substance delivery carriers. Recently, catechol nanodrug modification has emerged as one of the most powerful methods^[^ ^16^ ^]^ for upgrading exosomes, which are widely used to coat various surfaces, such as bacterium and cells, to achieve a controllable drug delivery.

Exosomes are commonly applied to wound beds using the traditional peri‐wound injection method, which can lead to uneven drug distribution and rapid drug loss within a few hours, thereby affecting the treatment outcomes. This often necessitates repeated injections, resulting in inconvenience and extra treatment costs.^[^ ^17^ ^]^ When exosomes are encapsulated in an injectable hydrogel, uniform drug distribution can be achieved on the wound beds, along with reduced drug dosage, and facilitates slow and controlled release. Terefore, the exosome anchor peptide CP05^[^ ^18^ ^]^ enhanced injectable hydrogel enables a longer sustained drug release was applied in this study.

**Figure 1:**
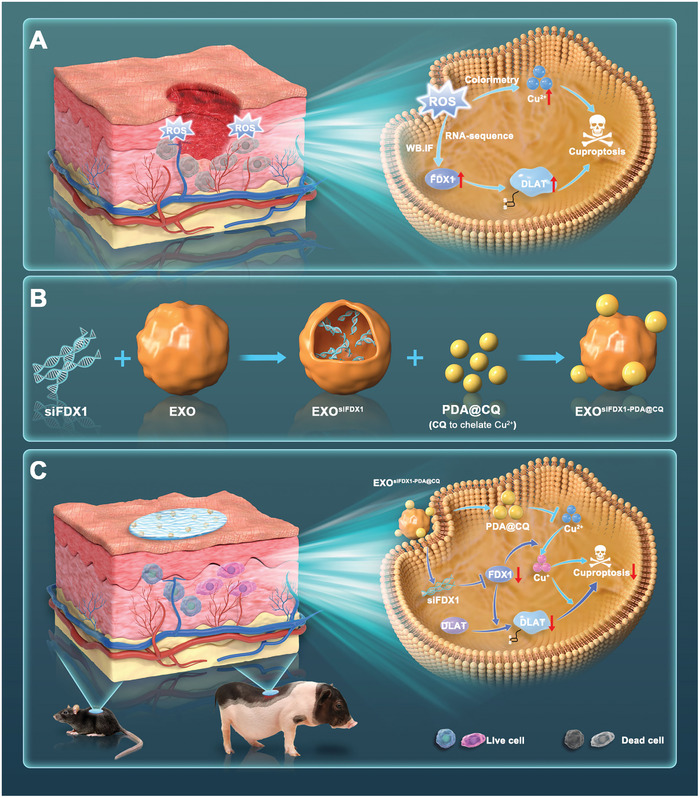
Schematic of the workflow for the therapeutic application of nanodrug‐modified engineered exosomes (EXO^siFDX1‐PDA@CQ^). EXO^siFDX1‐PDA@CQ^ releases drug‐chelated Cu through the breakdown of PDA particles after endocytosis and siRNA through exosome rupture. EXO^siFDX1‐PDA@CQ^ encapsulated in injectable exosomes with sustained release hydrogel exhibits strong efficacy in promoting wound healing by synergistically inhibiting the cuproptosis through reducing intracellular Cu content and reducing FDX1 expression.

In this study, as exhibited in **Figure** **1**A, the presence of FDX1‐mediated cuproptosis was confirmed in skin wounds and ROS‐induced cells model through colorimetric, RNA sequencing (RNA‐seq), and immunofluorescence experiments. Then, a specialized engineered exosomes polydopamine^[^ ^19^ ^]^ (PDA)@clioquinol^[^ ^20^ ^]^ (CQ) (EXO^siFDX1‐PDA@CQ^) was designed to target the dual‐pathway mechanism of cuproptosis, as depicted in Figure 1B. Briefly, CQ, an effective Cu‐ion chelating agent, can be conveniently loaded in PDA nanoparticle (NP)^[^ ^21^ ^]^ to develop a PDA@CQ nanodrug. PDA@CQ NPs are loaded onto the outer membrane of engineered exosomes through surface adhesion, covalent, and non‐covalent binding interactions, while the siRNA‐FDX1 within the engineered exosomes achieves a strong inhibition of FDX1 expression, addressing the issue of a single gene regulation mode for exosome delivery. To attain a longer drug‐retention time, the CP05‐enhanced injectable hydrogel is fabricated as a transportation facility to administer EXO^siFDX1‐PDA@CQ^. As illustrated in Figure 1C, the hydrogel EXO^siFDX1‐PDA@CQ^ was injected onto the wound beds of diabetic mice and pigs. Based on the results, the EXO^siFDX1‐PDA@CQ^ enhance cuproptosis inhibition effectiveness through the matched dual‐pathway approach, thereby promoting wound healing.

## 2 Results and Discussion

### 2.1 Cuproptosis in Wound Beds

**Figure 2:**
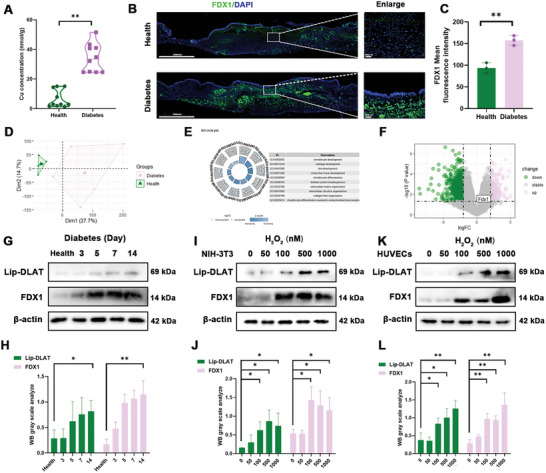
Cuproptosis hinders wound healing in diabetic wounds. A) Cu content in the healthy and diabetic mouse. B,C) Detection of FDX1 expression levels in healthy and diabetic mouse wound groups. FDX1 (green); DAPI (blue). Scale bar = 100 mm (left) or 20 µm (right). D) Results of PCA and DEGs analysis in diabetic and normal wounds. E) Enrichment analysis of 867 DEGs. F) The expression of FDX1 mRNA in volcano plot. G,H) Western blotting was used to detect the expression of the cuproptosis protein in healthy and diabetic wounds at different stages, and the results were analyzed. I–L) Western blotting was used to detect the expression of cuproptosis‐related proteins in the two types of wound cells under different H_2_O_2_ stimuli, and the results were analyzed. Data are expressed as mean ± SD. *p < 0.05, **p < 0.01. All data represent at least three independent experiments.

Previous studies have emphasized that oxidative stress exists in skin wounds and stimulates an increase in intracellular Cu.^[^ ^22^ ^]^ Excess Cu in cells causes an increase in Fe─S cluster proteins expression,^[^ ^23^ ^]^ leading to cuproptosis.^[^ ^9^ ^]^ To verify the aforementioned experimental results on skin wounds, colorimetry was used to detect the total Cu concentration in skin wounds tissues. As demonstrated in **Figure** **2**A, Cu content was significantly increased in diabetic wounds compared with healthy wounds.

Recently, the occurrence of cuproptosis has been reported to depend on Cu overload and is also closely related to FDX1 upregulation.^[^ ^9^ ^]^ There are no reports indicating the association between oxidative stress in diabetic wounds and cuproptosis caused by copper overload, and the specific role of FDX1 in cuproptosis in diabetic wounds is still unclear. Thus, immunofluorescence was conducted to evaluate the presence of FDX1 protein in diabetic mice. As shown in Figure 2B,C, diabetic wounds exhibit significantly higher FDX1 protein levels than that in healthy mice (*P* < 0.01). To further uncover the pathophysiology of diabetic wounds, RNA‐seq was performed on both diabetic and healthy wounds. Principal component analysis (PCA) results indicated that there are significant differences in gene expression between these two groups (Figure 2D). Differential expression gene (DEG) analysis revealed 508 downregulated and 359 upregulated genes in the diabetic group compared with the health group (Figure 2E). These downregulated genes are associated with skin, chondrocyte, and cartilage development, suggesting that the wound repair process is impaired in diabetic mice. Consistent with our immunofluorescence result, FDX1 serves as a crucial regulator of cuproptosis and it is significantly upregulated in diabetic wounds (*P* < 0.05) (Figure 2F).

The cuproptosis‐related proteins lipoylated Dihydrolipoamide S‐Acetyltransferase (Lip‐DLAT)^[^ ^9^ ^]^ and FDX1 were subsequently detected in wound samples from healthy and diabetic mice. The results were assessed using grayscale intensity analysis, which revealed that healthy mice wounds rarely express cuproptosis‐related proteins. However, as the duration of diabetes prolong, cuproptosis‐related proteins Lip‐DLAT and FDX1 expression increase significantly (*P* < 0.05), indicating that more cells undergo cuproptosis (Figure 2G,H). Fibroblasts (NIH‐3T3) and Human Umbilical Vein Endothelial cells (HUVECs) were used to establish cell models in vitro. Different concentrations of hydrogen peroxide (H_2_O_2_) were used to mimic oxidative stress in diabetic wounds at different periods. Western blotting was used to detect cuproptosis‐related proteins. The results showed that the expression level of cuproptosis related proteins increased synchronously with the increase of H_2_O_2_ concentration (Figure 2I–L). Moreover, we detected the expression level of FDX1 in two kinds of cells under different glucose concentrations. The Figure S1 (Supporting Information) manifested that with the increase of glucose concentration, the expression level of this gene also increased, and there was a statistical difference with the control group (*p* < 0.05). These findings suggest that both excessive ROS and high glucose levels contribute to a gradual increase in the expression levels of proteins associated with cuproptosis, which corroborates with our in vivo model.

### 2.2 Construction of Engineered Exosomes

**Figure 3:**
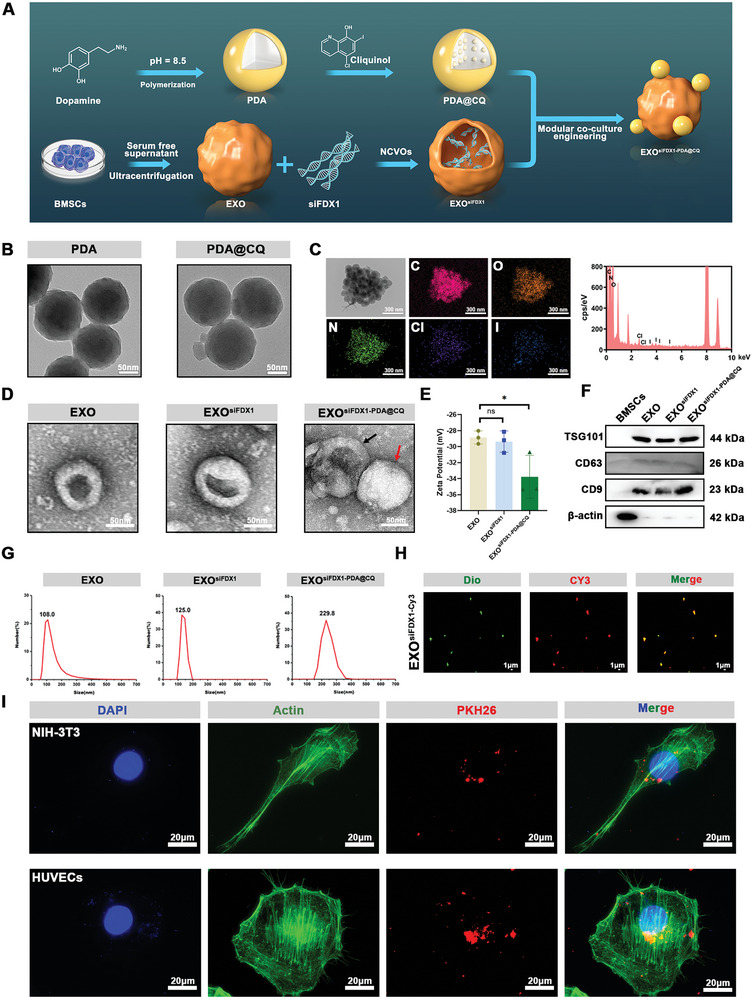
Constructing engineered exosomes modified with nanomedicine. A) Workflow of manufacturing EXO^siFDX1‐PDA@CQ^. B) Morphology of PDA and PDA@CQ determined by TEM. Scale bar = 50 nm. C) The content of different elements in PDA@CQ determined by TEM and energy dispersive spectroscopy. Scale bar = 300 nm. D) Images of three different modified exosomes captured by TEM (Black Arrow: Exosomes; Red Arrow: PDA@CQ). Scale bar = 50 nm. E) Measuring the surface Zeta potential of the EXO, EXO^siFDX1^, and EXO^siFDX1‐PDA@CQ^ groups. F) Detecting surface markers of BMSCs, EXO, EXO^siFDX1^, and EXO^siFDX1‐PDA@CQ^. G) Particle sizes measured by DLS. H) Fluorescence microscope images depicting the visual effect of siFDX1‐Cy3 (Red) encapsulated in Dio‐stained exosomes (Green). Scale bar = 1 µm. I) Fluorescence microscope images depicting the visual effect of PKH26‐stained EXO^siFDX1‐PDA@CQ^ (Red) successfully endocytosed by two types of wound cells. Scale bar = 20 µm. Data are expressed as mean ± SD. *P < 0.05, **P < 0.01. All data represent at least three independent experiments.

Engineered exosomes were designed as to effectively reduce wound cell death caused by cuproptosis. As illustrated in **Figure** **3**A, exosomes were harvested from bone marrow mesenchymal stem cells (BMSCs) using gradient centrifugation, followed by the internal loading of siRNA through a typical water bath ultrasonography technique. Simultaneously, a U.S. Food and Drug Administration‐approved hydrophobic chelating agent (CQ) of Cu was used and synthesized by a PDA‐based nanocarrier (PDA@CQ) via self‐polymerization of catechol and drug absorption methods for drug encapsulation and exosomal hitchhiking. Then, nanocontact vortex oscillators and modular co‐culture engineering were used to develop exosomes with nanomedicine, endowed with gene knockout capability (hence referred to as “EXO^siFDX1‐PDA@CQ^”). After being taken up by cells, these EXO^siFDX1‐PDA@CQ^ will initially release drugs from their internal mechanisms through the rupture of the PDA shell. Subsequently, exosome membrane decomposes, siRNA within the exosomes can be released and downregulate FDX1 expression.

As displayed in Figure 3B, the PDA NPs are spherical with a diameter of 120 nm. After encapsulating CQ into PDA to form PDA@CQ, there was no significant change in their particle size. Subsequently, the energy dispersive spectrometry confirmed the presence of I and Cl elements in the PDA@CQ NPs, which are unique to CQ, proving the successful encapsulation of CQ within PDA NPs (Figure 3C). The typical loading content of CQ in PDA NPs anchors on exosomes was calculated to be 1.2% (Figure S2A—C, Supporting Information). As displayed in Figure 3D, images were obtained by transmission electron microscopy (TEM), the morphology of siRNA‐loaded exosomes maintained a double concave disk shape without significant changes in particle size or membrane integrity compared with pure exosomes. From the TEM image, we can clearly observe the combination of exosome and PDA@CQ, and the particle size increased to 200 nm. The particle size, Zeta potential and surface marker expression of these nanoparticles were also examined.

The results indicated that the membrane surface potential of EXO^siFDX1‐PDA@CQ^ decreased from −27 to −34 mV compared to EXO, due to the presence of negatively charged PDA on its surface (Figure 3E). However, there was no difference in the surface marker of the EXO^siFDX1‐PDA@CQ^ compared with EXO or EXO^siFDX1^ (Figure 3F). In addition, DLS detection revealed that the particle size increased to 229.8 nm in the EXO^siFDX1‐PDA@CQ^, which was consistent with the TEM results (Figure 3G).

To confirm the successful incorporation of siFDX1 into the EXO^siFDX1‐PDA@CQ^, Cy3‐modified^[^ ^24^ ^]^ siRNA exhibiting red fluorescence and Dio‐stained^[^ ^25^ ^]^ exosomes displaying green fluorescence were utilized to assess their localization using a fluorescence microscope. As demonstrated in Figure 3H, significant colocalization of red and green fluorescence was observed under a fluorescence microscope, indicating the successful encapsulate of siRNA in EXO^siFDX1‐PDA@CQ^. To confirm that the EXO^siFDX1‐PDA@CQ^ can be absorbed by cells, they were labeled with PKH26 with red fluorescence and co‐incubated with cells for 24 h. The endocytosis of exosomes was then observed by staining the cytoskeleton and nucleus under a fluorescence microscope. Exosomes displaying red fluorescence were clearly observed within the green fluorescent cytoskeleton, indicating the successful internalization of EXO^siFDX1‐PDA@CQ^ by the cells (Figure 3I).

### 2.3 Construction of Hydrogel for Encapsulated EXO^siFDX1‐PDA@CQ^

**Figure 4:**
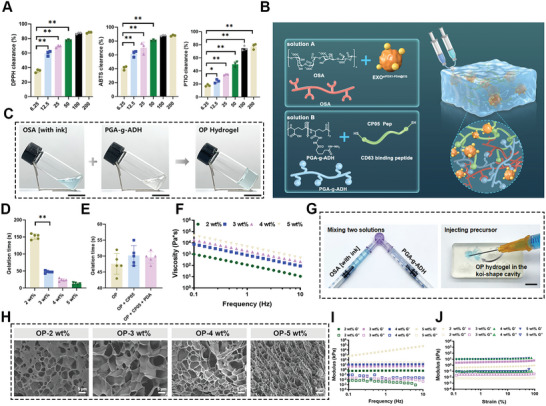
Fabrication and evaluation of hydrogel. A) DPPH, ABTS, and PTIO scavenging ability of PDA@CQ NPs. B) Schematic of the formation of PDA@CQ and gel network of OP hydrogel. C) Representative images of precursors and hydrogel fabrication process. Scale bar = 1 cm. D) Gelation time of various concentrations of OP hydrogel. E) Gelation time of various OP hydrogel. F) Evaluation of frequency‐responsive rheological characteristics of the customized hydrogel under a 1% strain at 37 °C. G) Injectability of OP hydrogel. Scale bar = 1 cm. H) Representative SEM images of different concentrations of OP hydrogels. Scale bar = 5 µm. I) Evaluation of frequency‐responsive rheological characteristics of the customized hydrogel under a 1% strain at 37 °C. J) Analysis of strain‐responsive rheological behavior of the customized hydrogel at a frequency of 0.1 Hz at 37 °C and gel strength of different hydrogel.

PDA NPs are well‐studied nanomaterials with excellent antioxidation properties. In this study, the ROS scavenging performance of the as‐prepared PDA@CQ NPs was elaborately evaluated using a semi‐quantitative spectroscopic analysis. **Figures** **4**A and S3 (Supporting Information) demonstrate that the three representative radicals, 2,2‐diphenyl‐1‐picrylhydrazyl (DPPH), 2,2‐binamine‐di‐3‐ethylbenzothiazolin‐6‐sulfonic acid (ABTS), and 2‐Phenyl‐4,4,5,5‐tetramethylimidazoline‐1‐oxyl 3‐oxide (PTIO), can be eliminated within 30 min of incubation with PDA@CQ NPs, and the ROS elimination rate is positively correlated with concentrations of PDA@CQ NPs. The concentration thresholds of 80% ROS clearance were 50 µg mL^−1^ for DPPH and ABTS radicals and 200 µg mL^−1^ for PTIO, indicating an efficient anti‐ROS performance of PDA@CQ NPs. Collectively, the as‐prepared PDA@CQ NPs with antioxidation and potential Cu‐ion chelation can be a potent participant in exosome surface engineering.

Subsequently, two biomacromolecules of oxidized sodium alginate (OSA) and polyglutamic acid grafting adipic dihydrazide (PGA‐g‐ADH) were synthesized (Figures S4 and S5, Supporting Information). An injectable hydrogel (OP) encapsulating EXO^siFDX1‐PDA@CQ^ was fabricated using a Schiff base “click” chemistry of OSA, PGA‐g‐ADH, and thiolated exosomal anchoring linkers (CP05‐Pep) to prolong the retention of EXO^siFDX1‐PDA@CQ^ in diabetic ulcers^[^ ^26^ ^]^ (Figure 4B). The as‐synthesized OSA polymer containing aldehyde groups facilitates rapid gelation with PGA‐g‐ADH and CP05‐Pep in ambient conditions^[^ ^27^ ^]^ (Figure 4C). The gelation time of the customized hydrogel was examined using the vial inversion method.^[^ ^28^ ^]^ The gelation velocity was positively correlated with the concentrations of two macromers. Intriguingly, we were unable to capture the solidification process when the concentrations of OSA and PGA‐g‐ADH were set to 1 wt%; however, the gelation process was significantly accelerated when the concentrations were increased to 2 wt% and 3 wt% (Figure 4D). Moreover, the addition of CP05 Pep and PDA NPs had a minimal impact on gelation time, highlighting the major contributions of the Schiff base reaction between OSA and PGA‐g‐ADH in the curing process (Figure 4E). The resulting OP‐3 wt% hydrogel was stably cured within 50 s, with no visible leakage. Considering the ease of use, 3 wt% was selected as the optimal concentration for subsequent investigations. To detect the distribution of EXO^siFDX1‐PDA@CQ^ in the hydrogel, they were labeled with PKH26 red fluorescence. As demonstrated in Figure S6 (Supporting Information), PKH26 labeled exosomes were evenly distributed in the hydrogel, indicating successful encapsulation of exosomes. Additionally, the viscosity of hydrogel declined markedly with the increase in the shear rate, suggesting a typical shear‐thinning behavior to provide convenience for injection (Figure 4F). The modular in situ gelling behavior of the two solutions allows the hydrogel to have excellent injectability and conveniently extrude from the needle of a disposable syringe as depicted in Figure 4G, indicating great facilitation in clinical surgical operations. As displayed in Figure 4H, the porosity of gel frameworks increased with increasing precursor concentration, indicating a denser structure. To evaluate the stability of the as‐fabricated hydrogels under dynamic conditions, their rheological properties were examined using frequency‐ and strain‐responsive sweep tests. Various concentrations of hydrogel behaved stably at a range of frequencies (0.1–10 Hz, Figure 4I) and strains (0.1%–100%, Figure 4J). Furthermore, the addition of PDA NPs into the gel blend had a negligible influence on the rheological properties of the pristine OP hydrogel (Figure S7A—D, Supporting Information). As a result, the gel strength of the OP‐3 wt% hydrogel was determined as 5 kPa (Figure 4J). Due to the reversible nature of the chemical linkages, the as‐fabricated OP hydrogel self‐healed under alternative strains without compromising gel strength (Figure S7E, Supporting Information), demonstrating a suitable mechanical performance as a wound dressing and remarkable adaptation to physiological dynamics. The successfully synthesized hydrogel was used to cover the skin of mice and pigs, and its adhesion was tested by squeezing, twisting, inverting, and adding water. The aforementioned experimental results demonstrated that the adhesive can adhere effectively to the skin of mice (Figure S8, Supporting Information) and pigs (Figure S9, Supporting Information). As illustrated in Figure S10 (Supporting Information), the engineered exosomes‐laden hydrogel also possess excellent ROS scavenging ability (detailed information in Supporting Information).

Before in vivo administration, the hemocompatibility of the resulting OP hydrogel was evaluated. The anti‐hemolysis characteristics of the as‐prepared hydrogels at various concentrations were simultaneously investigated (Figure S11, Supporting Information). Triton X‐100 was used as a positive control to completely hemolyze the diluted blood. Therefore, the hemolysis rate of various kinds of hydrogels with increasing concentrations was calculated to be less than 0.5%, indicating an excellent hemocompatibility of the resultant hydrogels.

### 2.4 EXO^siFDX1‐PDA@CQ^ Synergistic Inhibition of Cuproptosis

**Figure 5:**
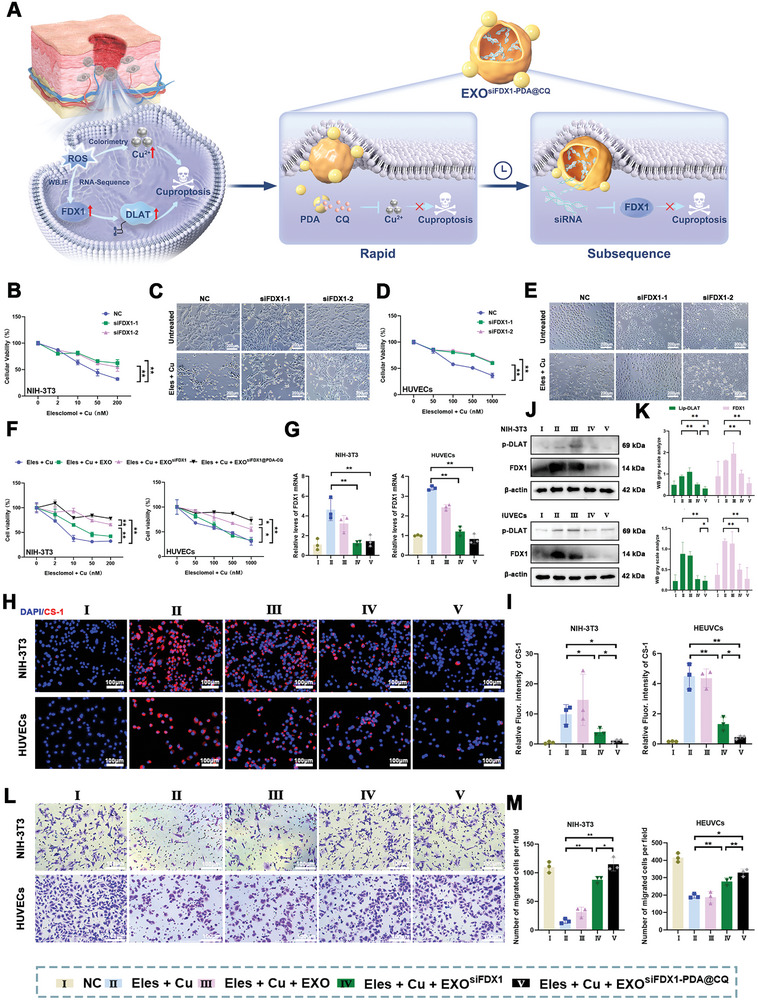
EXO^siFDX1‐PDA@CQ^ pathway synergistically inhibits cuproptosis in both space and time. A) Schematic of the mechanism of the synergistic inhibition of cuproptosis by EXO^siFDX1‐PDA@CQ^. B–E) The CCK8 assay was used to detect the occurrence of cuproptosis in cells after knockdown with siRNA, and morphological changes were observed under light microscopy. Scale bar = 200 µm. F) Extent of cell viability improvement after treatment with EXO^siFDX1‐PDA@CQ^. G) qRT‐PCR was used to detect the degree of downregulation of FDX1 in cells after treatment with EXO^siFDX1‐PDA@CQ^. H,I) Fluorescence microscopy was used to detect intracellular Cu^+^ concentrations subjected to different treatments. Scale bar = 100 µm. (J,K) Western blotting was used to detect the expression of cuproptosis‐related proteins in cells after treatment with EXO^siFDX1‐PDA@CQ^, and statistical analysis was performed. (L,M) Transwell assay was used to detect the migration ability in each group. Scale bar = 100 µm. Data are expressed as mean ± SD. *P < 0.05, **P < 0.01. All data represent at least three independent experiments.

EXO^siFDX1‐PDA@CQ^ may achieve dual synergy at the spatiotemporal and molecular levels by simultaneous two‐pathway inhibition of cuproptosis (**Figure** **5**A). Subsequently, two different cell types cuproptosis models were constructed using Elesclomol (Eles, a Cu^2+^ ionophore known for its role in cuproptosis) and cupric chloride (CuCl_2_). These cuproptosis models demonstrated that FDX1 downregulation significantly inhibited cell death (*P* < 0.05) (Figure 5B,D) while maintaining normal cell morphology (Figure 5C,E). A general validation of the cell survival rate using CCK8 and cell viability staining was performed to verify the function of EXO^siFDX1‐PDA@CQ^. The results indicated that both EXO^siFDX1^ and EXO^siFDX1‐PDA@CQ^ effectively inhibit cell death, with EXO^siFDX1‐PDA@CQ^ exhibiting a better effect than EXO^siFDX1^ (*P* < 0.05) (Figure 5F; Figure S12, Supporting Information). Intracellular FDX1 levels increased after the addition of Eles and CuCl_2_. Additionally, EXO^siFDX1^ and EXO^siFDX1‐PDA@CQ^ treatment effectly knockdown the FDX1 mRNA expression (*P* > 0.05) (Figure 5G), indicating that PDA@CQ on the surface of exosomes does not affect the function of internal siRNA. In contrast, the CS‐1 probe was performed to detect intracellular Cu^+^ levels. Statistical analysis of their fluorescence intensity revealed that downregulating FDX1 can inhibit the transformation of partially overloaded Cu^2+^ into Cu^+^, but this impact is significantly enhanced by adding PDA@CQ (*P* < 0.05) (Figure 5H‐I). Then, both of the Western blotting and qRT‐PCR results demonstrated the successful downregulation of FDX1 expression level. These results indicated that both EXO^siFDX1^ and EXO^siFDX1‐PDA@CQ^ are capable of efficiently downregulating FDX1. Moreover, the EXO^siFDX1‐PDA@CQ^ had better Lip‐DLAT downregulation effect than EXO^siFDX1^ (*P* < 0.05) (Figure 5J,K). Moreover, to assess whether cuproptosis inhibition can restore cell migration ability, we conducted cell transwell experiments. Based on our results, the cells in the EXO^siFDX1‐PDA@CQ^ group exhibited the best cell migration ability compared to the other groups (Figure 5L,M).

In addition, we also detected the migration effect of our EXO^siFDX1‐PDA@CQ^ on NIH‐3T3 cells and HUVECs cells in the H_2_O_2_ induced cuproptosis model. We found that EXO^siFDX1‐PDA@CQ^ could restore the migration ability of cells, and compared with H_2_O_2_ group and H_2_O_2_ + EXO^siFDX1^ group. The scratch area of cells in EXO^siFDX1‐PDA@CQ^ group was the smallest and the most pronounced difference was observed at 48 h (*P* <0.05) (Figure S13, Supporting Information). The tube forming experiment against HUVECs also showed that EXO^siFDX1‐PDA@CQ^ could restore the tube forming ability of cells (Figure S14, Supporting Information). Besides, qRT‐PCR results suggests that after EXO^siFDX1‐PDA@CQ^ treatment, the VEGFA (Vascular endothelial growth factor A) mRNA level of HUVECs and Col‐I (Collagen Type I) mRNA level of NIH‐3T3 cells were the highest (*P* <0.05) (Figure S15, Supporting Information).

### 2.5 EXO^siFDX1‐PDA@CQ^ Promotes Wound Healing

**Figure 6:**
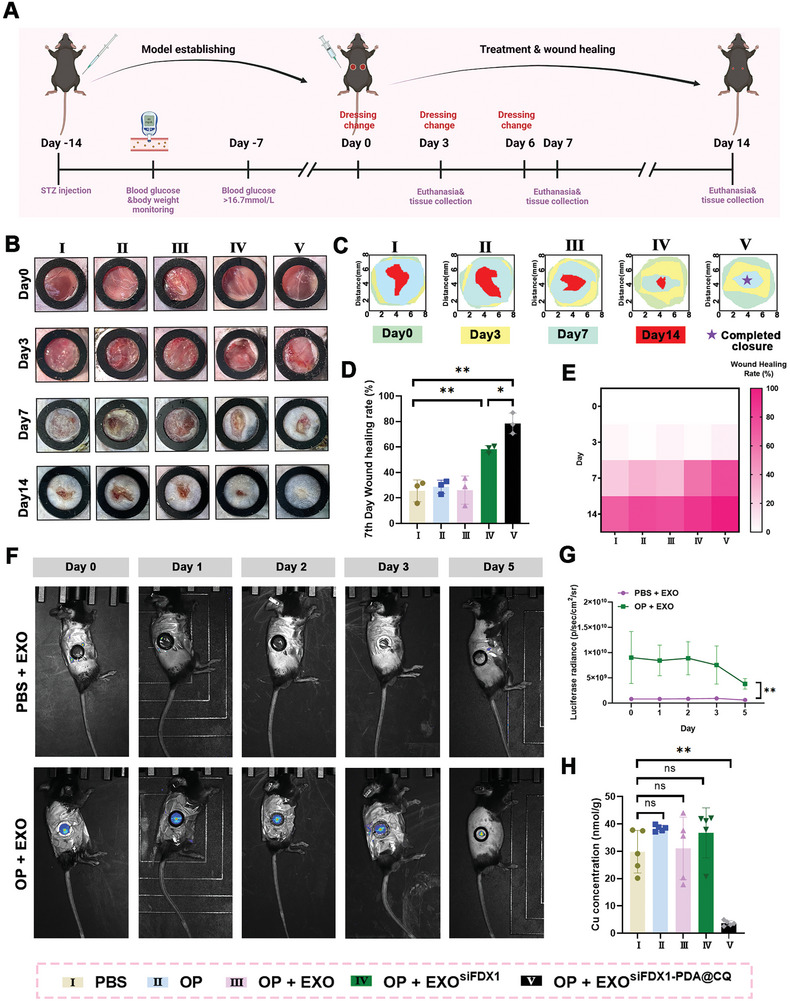
EXO^siFDX1‐PDA@CQ^ effectively promotes diabetic wound healing. A) The schematic illustrates the establishment and treatment of a diabetic wound mouse model (created by BioRender.com). B) representative images of each group of diabetic wounds during the healing process. C) Simulation diagram of wound closure area. D) Statistical analysis of wound healing rate in different groups on the 7th day. E) Statistics of wound healing rate over time in 0–14 days. F,G) In vivo imaging was used to determine the survival time and content of PKH26 labeled EXO^siFDX1‐PDA@CQ^ after injection and OP hydrogel administration. H) The Cu content in tissues was detected using colorimetry. Data are expressed as mean ± SD. *P < 0.05, **P < 0.01. All data represent at least three independent experiments.

**Figure 7:**
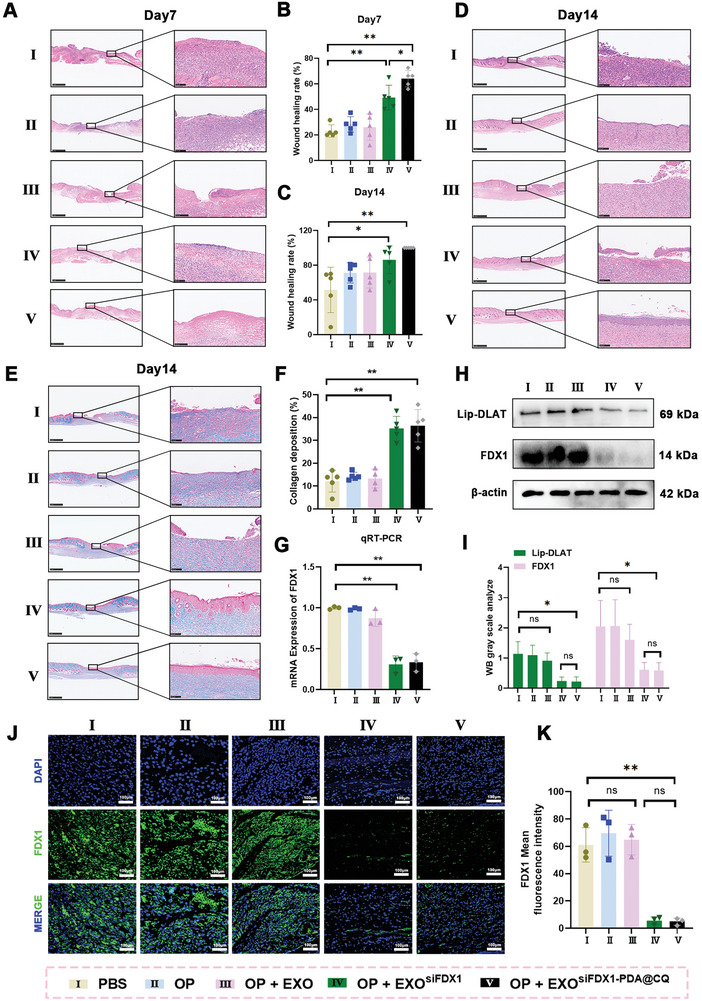
EXO^siFDX1‐PDA@CQ^ accelerates the healing of diabetic wounds by reducing Cu content and inhibiting the expression of FDX1. A–D) H&E staining images of wound samples from different groups were quantified on days 7 and 14, and wound length was quantified. Scale bar = 1 mm (left) or 100 µm (right). E,F) Masson staining images and statistical collagen content of the wound samples in different groups on the 14th day after surgery. Scale bar = 1 mm (left) or 100 µm (right). G) FDX1 mRNA levels in different stem groups were determined using qRT‐PCR. H,I) Western blotting analysis of Lip‐DLAT and FDX1 expression level. J,K) FDX1 expression images of different groups of tissues under a fluorescence microscope. FDX1 (green); DAPI (blue). Scale bar = 100 µm. Data are expressed as mean ± SD. *P < 0.05, **P < 0.01. All data represent at least three independent experiments.

To evaluate the efficacy of EXO^siFDX1‐PDA@CQ^ in vivo, streptozotocin (STZ) was intraperitoneally injected to establish a typical I diabetic mouse model.^[^ ^29^ ^]^ A consecutive reading exceeding 16.7 mmol L^−1^ indicates the successful establishment of the diabetic model. Additionally, the weight of mice (Figure S16A, Supporting Information) and blood glucose levels (Figure S16B, Supporting Information) were regularly monitored. The results indicated that weight decreased with time, although there was no statistically significant difference between the various groups. Subsequently, a round full‐thickness skin wound with an 8‐mm diameter was created on the back of mice (**Figure** **6**A). Wound closure rate was analyse using ImageJ software (NIH, ImageJ 1.8, USA). As depicted in Figure 6B, on the 14th day the wound area in the OP + EXO^siFDX1‐PDA@CQ^ group healed and was covered by new epithelial cells, whereas the other four groups still exhibited open wounds and scabs. This result was statistically analyzed and is evident on the wound area map in Figure 6C. On the 7th day, it was observed that the healing rates were not significantly different between the PBS, OP, and OP + EXO groups. The healing rate of the OP + EXO^siFDX1^ group was significantly higher than other PBS, OP, and OP+EXO groups (*P* < 0.01), and the OP + EXO^siFDX1‐PDA@CQ^ group exhibited a highest healing rate (*P* < 0.05) (Figure 6D). Moreover, according to the analysis of 0 to14 wound healing rate, EXO^siFDX1‐PDA@CQ^ group showed the best effect among the five groups (Figure 6E). A comparative analysis of the retention rates between injections and OP capsules incorporating EXO^siFDX1‐PDA@CQ^ was performed to validate drug retention using the IVIS Lumina Series. According to the results, OP exerted a better‐sustained release effect on EXO^siFDX1‐PDA@CQ^ than the injection group, with the effect lasting up to three days. On the fifth day, the exosome content on the surface of the wound significantly decreased. Therefore, we change the dressing on the 3rd and 6th day (Figure 6F,G). Moreover, the colorimetric method was also used to detect the Cu concentration in wound tissues. The results demonstrated that only EXO^siFDX1‐PDA@CQ^ administration can reduce the Cu level (*P* <0.01) (Figure 6H). As displayed in **Figure** **7**A–D, the wound length in the OP + EXO^siFDX1^ group was significantly shorter than that in the PBS, OP, and OP + EXO groups (*P* < 0.01). Similarly, the OP + EXO^siFDX1‐PDA@CQ^ group exhibited a higher wound healing rate than the OP + EXO^siFDX1^ group (*P* < 0.05). As seen in Masson staining (Figure 7E,F), the OP + EXO^siFDX1‐PDA@CQ^ group exhibited notably increased collagen deposition and a more organized pattern of fibroblast alignment than the PBS, OP, OP + EXO, and OP + EXO^siFDX1^ groups. In summary, EXO^siFDX1‐PDA@CQ^ effectively promotes diabetic wound healing.

To further verify the in vivo inhibitory effects of EXO^siFDX1‐PDA@CQ^, FDX1 mRNA levels were evaluated using qRT‐PCR (Figure 7G). The results indicated that both EXO^siFDX1^ and EXO^siFDX1‐PDA@CQ^ successfully downregulated the mRNA level of FDX1 (*P* < 0.01). Besides, Western blot result showed that the expression of FDX1 and Lip‐DLAT was significantly reduced (Figure 7H,I). At the same time, immunofluorescence experiments also proved that the expression of FDX1 was significantly downregulated in EXO^siFDX1^ and EXO^siFDX1‐PDA@CQ^ groups (*P* <0.01) (Figure 7J,K). Furthermore, the Lip‐DLAT expression level was considerably‌ decreased in EXO^siFDX1‐PDA@CQ^ groups demonstrated that cuproptosis was significantly inhibited. These findings were consistent with in vitro experimental results. Additionally, hematoxylin and eosin (H&E) staining was performed on key organs in each group, including the heart, liver, spleen, lungs, and kidneys. The results indicated that neither the hydrogel nor the EXO^siFDX1‐PDA@CQ^ caused any evident damage to these organs (Figure S17, Supporting Information), indicating excellent tissue biocompatibility.

### 2.6 EXO^siFDX1‐PDA@CQ^ Promotes Wound Healing in a Full‐Thickness Pig Skin Wound Model

**Figure 8:**
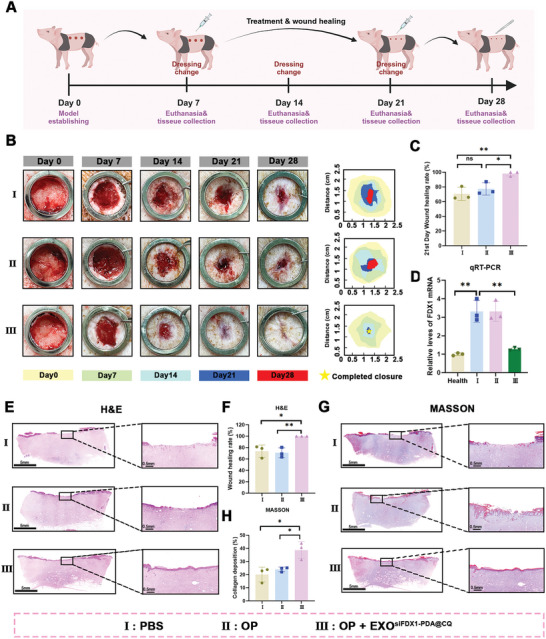
Functional validation of engineered exosomes modified with nanomedicines in a full‐thickness pig skin wound model. A) The schematic illustrates the establishment and treatment of a full‐thickness pig skin wound model (created by BioRender.com). B) Representative images of wounds in each group during healing. C) Quantitative assessment of wound closure rate on postoperative day 21. D) Detection of FDX1 mRNA expression levels in healthy skin and wound tissues in each group. E,F) H&E staining images of wound samples from different groups on postoperative day 21, and the wound length was quantified. Scale bar = 5 mm (left) or 0.5 mm (right). G,H) Masson staining images of the wound samples from different groups on postoperative day 21, and the quantitative analysis of wound collagen deposition. Scale bar = 5 mm (left) or 0.5 mm (right). Data are expressed as mean ± SD. *P < 0.05, **P < 0.01. All data represent at least three independent experiments.

Due to the similar wound healing processes in pigs and humans, we extended our exploration into the efficacy of EXO^siFDX1‐PDA@CQ^ to a full‐thickness pig wound model (**Figure** **8**A). Repairing large skin defects, as opposed to small wounds, requires longer granulation tissue formation and maturation time to cover the wound, which involves a variety of growth factors and cell interactions.^[^ ^30^ ^]^ Any dysregulated event during the wound‐healing process may lead to the refractory wounds. Large wounds have an immune microenvironment barrier; thus, a full‐thickness skin defect wound with a diameter of 2 cm was established on the backs of pigs. The digital photos of wound in each group demonstrated that, wound healing rate was highest in OP + EXO^siFDX1‐PDA@CQ^ group compared with the PBS group OP groups (Figure 8B). And the most significant effect was observed on the 21st day (*P* <0.05) (Figure 8C). Subsequently, on the 21st day, wounds and normal skin tissue samples were collected, and RNA was extracted. According to the qRT‐PCR results, FDX1 expression levels were significantly higher in wounds compared to normal skin (*P* < 0.01), and the FDX1 expression levels were significantly downregulated by the administration of EXO^siFDX1‐PDA@CQ^ (*P* < 0.01) (Figure 8D). By the 28th day post‐surgery, the OP + EXO^siFDX1‐PDA@CQ^ group exhibited nearly complete wound healing, contrasting with the OP and PBS groups, where the wounds remained open with visible scabs. As illustrated in Figure 8E,F, there was no significant difference in wound length between the OP and PBS groups on day 28. However, the wound length in the OP + EXO^siFDX1‐PDA@CQ^ group was significantly shorter than that in the OP and PBS groups, with nearly complete re‐epithelialization of wounds (*P* < 0.05). Meanwhile, statistical analysis indicated no significant difference in collagen deposition between OP and PBS groups on day 28. The OP + EXO^siFDX1‐PDA@CQ^ group exhibited a markedly higher level of collagen deposition (*P* < 0.05) (Figure 8G,H). The experimental findings confirmed that EXO^siFDX1‐PDA@CQ^ exhibited exceptional efficiency and superior quality in facilitating wound healing, highlighting its promising therapeutic potential for clinical applications.

This study designed an engineered exosome, focusing on inhibiting cuproptosis through dual‐pathway synergistic action. Exosome surface nano‐drug modification is used to achieve intracellular copper chelation, immediately downregulating Cu^2+^, and combining it with intracellular siRNA interference to further downregulate the expression of cuproptosis key proteins FDX1. Molecular pathways and spatiotemporal coordinated intervention can maximize cuproptosis inhibition, which promote wound healing. However, due to limited specimen sources, this study only used animal models to verify the presence of cuproptosis in their wounds and the efficacy of intervention in improving refractory healing issues. The incidence and specific mechanisms of cuproptosis in human skin wounds and the relevant efficacy of this intervention method remain to be further verified.

## 3 Conclusion

In summary, these results indicate the presence of cuproptosis in refractory wounds, which is mediated by both Cu overloading and FDX1 upregulation pathways. The EXO^siFDX1‐PDA@CQ^ designed in this study can release CQ to chelate Cu ions in cells, resulting in the immediate inhibition of cuproptosis. Subsequently, internal siRNA is released to interfere with mRNA transcription and translation, preventing cuproptosis from occurring in the long term by downregulating FDX1 expression. This strategy effectively inhibits cuproptosis and promotes wound healing by combining dual pathways and dual temporal nanomedicine‐modified engineered exosomes. Given the presence of cuproptosis in refractory wounds, combining these engineered exosomes with a sustained‐release hydrogel may become a feasible and useful treatment strategy for for the management of clinically refractory wounds.

## 4 Experimental Section

### Cells and Culture Conditions

NIH‐3T3 and HUVEC (Culture Preservation Center of the Chinese Academy of Sciences, Shanghai, China) were cultured in high‐glucose Dulbecco's modified Eagle medium (DMEM, Pricella) supplemented with 10% Fetal Bovine Serum (FBS, Pricella) and 1% Penicillin and Streptomycin (Gibco). Cell line identification was performed using NIH‐3T3 and HUVECs. All cell lines were cultured in a humidified incubator at 37 °C with 5% CO_2_. Cells were studied in vitro using various concentrations of engineered exosomes.

### Source of Wound Tissue and Detection of Tissue Cu and mRNA Levels

Tissue was obtained from the wound sites of mice with type I diabetes. Wounds from normal and diabetic mice were collected over three days for subsequent examinations. RNA‐seq analysis (Novogene, China) was performed on the relevant tissues to evaluate Cu levels.^[^ ^31^ ^]^ A Cu microplate assay kit was acquired from Absin Bioscience (Shanghai, China). The liquid samples were wound tissue lysates extracted from 20 mice that were either healthy or had type I diabetes. The experiment was performed in accordance with the manufacturer's instructions.

### Immunofluorescence Analysis

To assess the expression of FDX1 in healthy and type I diabetic wound tissues, mouse skin samples were fixed for three days with 4% paraformaldehyde. They were then embedded in paraffin and sectioned. The samples were incubated overnight at 4 °C with a primary antibody against FDX1 (Abcam, USA). The next day, the sections were incubated with a secondary antibody (Boster, China) at room temperature before nuclear staining with DAPI (Yeasen, China) and were mounted. Images were captured using a fluorescence microscope, and three distinct samples were evaluated from each group. Finally, ImageJ software (NIH, ImageJ 1.8, USA) was used for analysis.

### Western Blotting

The tissue lysate was extracted using RIPA lysis buffer (Beyotime, China) containing 1% PMSF solution (Beyotime, China) and 1% protease inhibitor (Beyotime, China) at 4 °C for 20 min. Equal amounts of total protein (20–50 µg) were separated by SDS‐PAGE (Epizyme, China) and then separated with FDX1 (Abcam, USA), lipoic acid (Abcam, USA), and β‐actin (Proteintech, China). The membrane was incubated with a secondary antibody (Boster, China) and then exposed to an X‐ray film (UVP, USA). Imprint analysis was performed using ImageJ software (NIH, ImageJ 1.8, USA).

### High‐Throughput Sequencing

The analysis was conducted using Novogene software (Beijing, China). Briefly, wound tissues from type I diabetic mice, healthy mice, and normal skin were collected, immediately frozen in liquid nitrogen, and stored at –80 °C. The mRNA was then purified from total RNA using magnetic beads linked with poly (T) oligomers. The library fragments were purified using the AMPure XP system to preferentially select cDNA fragments ranging in length from 370 to 420 bp. Then, qRT‐PCR was performed using Phusion high‐fidelity DNA polymerase, universal qRT‐PCR primers, and Index (X) primers. RNA integrity was assessed using the RNA Nano 6000 assay kit on the Bioanalyzer 2100 system (Agilent Technologies, California, USA). TruSeq PE Cluster Kit v3 cBot HS (Illumina) was used to cluster index‐encoded samples on the Bot Cluster generation system, according to the manufacturer's instructions. Following cluster generation, the library was sequenced using the Illumina NovaSeq platform to generate 150 bp paired‐end reads.

### RNA‐seq and Bioinformatics Analyses

Bioinformatics analyses of the RNA‐seq data were conducted using R software (version 4.0.3). “FactoMineR” and “factoextra” packages were used for PCA. DEGs were analyzed using the “Limma” package (FDR < 0.05, |logFC| > 2). The “ClusterProfiler” package was used for enrichment analysis.

### Isolation and Characterization of BMSCs

Following euthanasia, the mice were immersed in 75% ethanol, and the tibia and femur were extracted under sterile conditions,^[^ ^32^ ^]^ including both ends of the tibia and femoral epiphysis. After washing the bone marrow cavity with PBS, the cell suspension was blown to allow the cells to flow out, which was repeated thrice. The bone marrow cell suspension was centrifuged at 1000 r min^−1^ for 5 min, the supernatant was removed, and the cell pellet was resuspended. This process was repeated thrice. The cell suspension was mixed with low glucose DMEM containing 15% FBS (Pricella, China) and inoculated into cell culture flasks. The cultivation environment was a sterile setting at 37 °C, which is conducive to adherent growth.

### Extraction of BMSCs‑Derived Exosomes

BMSCs‐derived exosomes (EXO) were isolated from FBS‐free BMSCs supernatant using differential centrifugation following a previously described protocol.^[^ ^33^ ^]^ The supernatant was centrifuged at 300 ×g for 10 min, 2000 × g for 10 min, and 10 000 × g for 30 min at 4 °C to remove cell debris. The sediment at the bottom of the tube was discarded, and the supernatant was collected and centrifuged at 100 000 × g for 70 min to extract exosomes. Extracellular vesicle microspheres were washed three times with sterile PBS and stored at −80 °C for later use.

### Synthesis of PDA@CQ

PDA NPs were fabricated using a typical self‐polymerization method.^[^ ^34^ ^]^ Briefly, 18 mg of dopamine was sufficiently dissolved in 9 mL of DI water. The polymerization was triggered under alkaline conditions (84 µL 1 M NaOH) at 60 °C for 5 h. After centrifugation and three washes with MeOH, the purified PDA NPs were dispersed in MeOH, and 5 mg CQ was added to the suspension. After 24 h of stirring under ambient conditions, the PDA@CQ NPs were collected via centrifugation and follow‐up vacuum drying. The morphologies of the PDA NPs and PDA@CQ NPs were assessed by TEM. The DPPH, ABTS, and PTIO radical scavenging capability of PDA NPs was tested via UV‐Vis spectroscopy.

### Ultrasonic Technology

Utilizing the ultrasound system from Sonic & Materials, Inc. (Newtown, USA), siFDX1 or siRNA‐Cy3 were loaded onto EXOs. EXOs and siFDX1 (Table S1, Supporting Information) were mixed in a 1:1 (mass/mass) ratio in PBS, resulting in a final EXO concentration of 4 µg mL^−1^. The ultrasound settings used were 20% amplitude, six 30‐sec on/off cycles with a 2‐min cooling period between each cycle.^[^ ^35^ ^]^ Following the ultrasonic treatment, the unbound siFDX1 in the supernatant was assessed at 260 nm using a microplate reader. The amount of siFDX1 in the supernatant was determined by the weight ratio of the encapsulated siFDX1 to the loaded siFDX1 nanovesicles. The solution was then incubated at 37 °C for 60 min to recover the exosome membrane and then maintained at 4 °C whenever possible before use. To confirm the successful encapsulation of siFDX1 by the exosome membrane, the exosomes were stained with Dio and loaded with siFDX1‐Cy3. After ultrasound incubation, the colocalization of Cy3 and Dio were observed under a fluorescence microscope.

### Nanocontact Vortex Technology

A suspension was prepared by adding PBS to EXO^siFDX1^ exosomes. The diluted suspension was filtered through a 0.22 µm filter for sterilization. Then, poly PDA‐CQ solution (0.5 mg mL^−1^) was prepared using distilled water. The PDA‐CQ solution was added to the mixture containing EXO^siFDX1^ exosomes in a 2:1 ratio (mass/mass) and mixed by vortex. EXO^siFDX1‐PDA@CQ^ suspension was prepared and incubated at 37 °C for 24 h. Then, 50 000 x g ultracentrifugation was performed for 70 min to remove free PDA‐CQ particles. The separated exosomes were stored at –20 °C for further analysis. The morphology of EXO^siFDX1‐PDA@CQ^ was analyzed using TEM. The particle size and zeta potential of the exosomes were determined using Dynamic Light Scattering (DLS, Malvern Panalytical, UK). The loading of CQ on EXO was verified using a UV‐Vis spectrometer.

### The Function of EXO^siFDX1‐PDA@CQ^ Complex In Vitro

A cuproptosis cell model was prepared using Eles (MedChemExpress, USA) and CuCl_2_ (Cu, Sigma Aldrich, USA). The experimental groups included the control, Eles + Cu, Eles + Cu + EXO, Eles + Cu + EXO^siFDX1^, and Eles + Cu + EXO^siFDX1‐PDA@CQ^ groups. The cell proliferation and migration ability were measured, and the intracellular Cu^+^ level was evaluated using Cu ion probe CS‐1 (MedChemExpress, USA). The results were observed and photographed using a fluorescence microscope. When the confluence of NIH‐3T3 and HUVECs reached 30%–50%, the EXO^siFDX1‐PDA@CQ^ (5 µg/well) complex was added. After incubation, images were randomly taken and observed.

A cuproptosis cell model was prepared using H_2_O_2_ (Sigma Aldrich, USA). The experimental group includes a control group, H_2_O_2_, H_2_O_2_ + EXO, H_2_O_2_ + EXO^siFDX1^, and H_2_O_2_ + EXO^siFDX1‐PDA@CQ^ Group, measure cell migration and tube forming ability.

### qRT‐PCR

Total RNA was isolated using an ultrapure RNA kit (Yeasen, China) according to the manufacturer's instructions. Total RNA was reverse transcribed into cDNA using PrimeScript RT kit (Yeasen, China). Then, qRT‐PCR was performed on the StepOne platform (Yeasen, China). See Table S2 (Supporting Information) for specific primer sequences.

### Fabrication and Evaluation of Hydrogel

The gel precursors of OSA and PGA‐g‐ADH were synthesized via chemical modification of natural biological macromolecules. Typically, 1 g of sodium alginate was dissolved in 100 mL of DI water, and then 0.6 g of NaIO_4_ was added into the solution for 24 h in the dark. Dialysis was conducted against DI water for three days, and the final OSA product was obtained by freeze‐drying. Then, 2 g of PGA (MW = 700 kDa) was dissolved in 200 mL of morpholine ethane sulfonic acid buffer (pH = 6.5). Afterwards, 4 g of 1‐(3‐dimethylaminopropyl)‐3‐ethyl carbodiimide hydrochloride and 1.8 g of N‐hydroxysuccinimide were added to the PGA solution and stirred for 30 min. Then, 9 g of ADH was added to the mixed solution for another 24 h. The reaction product was dialyzed against DI water for three days before freeze‐drying to obtain PGA‐g‐ADH. The successful synthesis of the as‐prepared OSA and PGA‐g‐ADH macromers was verified via ^1^H NMR, FTIR, GPC, and Zeta potential experiments.

A series of hydrogels was fabricated by mixing two precursors. In general, EXO^siFDX1‐PDA@CQ^ (2 µg mL^−1^) was dissolved in OSA‐6 wt% solution to obtain solution A. Solution B was prepared by mixing 6 wt% PGA‐g‐ADH and CP05‐polypeptide (CP05‐Pep) (2 µg mL^−1^). After that, gelation of the hydrogel was carried out spontaneously when the precursor solutions A and B were mixed in an equal volume. The gelation time of the customized hydrogel was measured by a vial inversion method. The dynamic rheological properties of various hydrogel samples were measured using a rheometer. For the determination of the strain‐responsive rheological behavior, the frequency was fixed at 0.1 Hz over the strain range from 0.1% to 100%. The gel strength was calculated based on the strain‐sweep tests done before. Frequency‐responsive rheological characteristics were tested at 1% strain over a frequency range of 0.1–10 Hz. The shear‐thinning behavior of hydrogel was verified in the viscosity mode while the shearing rate varied from 0.1 to 100 Hz. The temperature of the whole process of rheological measurements was set at 37 °C. The ROS scavenging performance of hydrogel was investigated using the aforementioned methods. The porous structure of the freeze‐dried hydrogel was observed via scanning electron microscopy (SEM). The hemolysis assay was used to evaluate the blood compatibility of the customized hydrogel. Whole blood from healthy swine was collected. Afterward, different hydrogels with various concentrations were co‐incubated with 4% diluted blood at 37 °C for 120 min. The hemolysis rate was quantified by measuring the absorbance value of the supernatant at 540 nm using a microplate reader (MD SoftMax Pro 5). Normal saline and Triton X‐100 were used as negative and positive controls, respectively.

### Animals’ Experiments

All animal experiments were approved by the Experimental Animal Ethics Committee of the Second Affiliated Hospital of Zhejiang University (No. 2024–137). For animal experiments, Six‐week‐old male C57BL/6 mice were intraperitoneally injected with 50 mg kg^−1^ STZ daily for five consecutive days. One week later, blood glucose was measured using a blood glucose monitor, and a blood glucose level above 16.7 mm indicated successful induction of diabetes. Mice were anesthetized with sodium pentobarbital (Sigma‐Aldrich) at 1% (50 mg kg^−1^), after which the dorsal hair was removed. A circular full‐thickness skin wound with a diameter of 8 mm was created following disinfection. The pressure applied to the wound was used to effectively control bleeding and further minimize hemorrhaging. Subsequently, a silicone ring was affixed to the back skin of mice using adhesive to prevent skin retraction. Mice were categorized into the control (PBS), OP (hydrogel only), OP + EXO (simple exosomes), OP + EXO^siFDX1^ (4 µg/wound, exosomes equipped with siFDX1), and OP + EXO^siFDX1‐PDA@CQ^ (4 µg/wound, exosomes equipped with siFDX1 and PDA@CQ) groups. Finally, the wound was covered with vaseline gauze (3 M, Tegaderm, USA) and a transparent dressing, which was changed on postoperative days 3 and 6. Photographs were taken on postoperative days 0, 3, 7, and 14. The wound area was measured using ImageJ software (NIH, ImageJ 1.8, USA).

### Histological Analysis

On the 7th and 14th day after injury, the entire injury bed of mice in each group was histologically analyzed. The skin tissue samples of diabetic and normal mice were stored at room temperature for histological examination and Western blotting analysis at –80 °C. The wound tissue was fixed with 4% paraformaldehyde. After dehydration, the tissue was embedded in paraffin and cut into 8 µm thick longitudinal sections for staining. H&E staining was used to observe the re‐epithelialization rate of wound tissue, and the Masson staining method was used to observe the accumulation of collagen. Additionally, the immunofluorescence was used to observe FDX1 expression.

### Small Animal In Vivo Imaging

A circular full‐thickness skin wound with a diameter of 8 mm was established, as described above. Mice were divided into injection (injection of exosomes with PKH26 fluorescence) and OP (hydrogel‐wrapped exosomes with PKH26 fluorescence) groups. Finally, the wound was covered with Vaseline gauze (3 M, Tegarderm, USA) and a transparent dressing. A live imaging device was used to take photos on postoperative days 0, 1, 2, 3, and 5 and to record the fluorescence retention of the wound.

### Pig Full‐Thickness Wound‐Healing Model

This study used Panamanian miniature pigs weighing 20–25 kg, with approval from the Animal Ethics Committee of the Second Affiliated Hospital of Zhejiang University School of Medicine (No. 2024–138). Following general anesthesia, the back hair of the miniature pigs was removed, and their backs were disinfected with iodine and 70% ethanol.^[^ ^36^ ^]^ Six circular full‐thickness skin wounds (diameter 2 cm, three on each side) were created and divided into three groups with a 4 cm distance between each group. After surgery, the wounds were randomly received PBS (control), OP (hydrogel only), and OP + EXO^siFDX1‐PDA@CQ^ (200 µg wound^−1^, exosomes equipped with siFDX1 and PDA@CQ), and all wounds were covered with transparent dressing (3M, Tegarderm, USA) immediately. A complete dressing change was performed on the 7th, 14th, and 21st days, consistent with the initial administration of the same therapeutic drug. Images were captured on postoperative days 0, 7, 14, 21, and 28 and analyzed using ImageJ software (NIH, ImageJ 1.8, USA).

### Statistical Analyses

The data is presented as mean ± standard deviation (SD). Statistical analyses were performed using GraphPad Prism 8.0 software (La Jolla, California, USA). The difference between the two groups was assessed using Student's t‐test. A one‐way analysis of variance with Tukey's multiple comparison test was used to compare multiple groups. Statistical significance was defined as *P < 0.05 or **P < 0.01, and ns indicates no statistical significance.

## Conflict of Interest

The authors declare no conflicts of interest.

## Supporting information



Supporting Information

## Data Availability

The data that support the findings of this study are available from the corresponding author upon reasonable request.

## References

[1] M. Zhao , M. Kang , J. Wang , R. Yang , X. Zhong , Q. Xie , S. Zhou , Z. Zhang , J. Zheng , Y. Zhang , S. Guo , W. Lin , J. Huang , G. Guo , Y. Fu , B. Li , Z. Fan , X. Li , D. Wang , X. Chen , B. Z. Tang , Y. Liao , Adv. Mater. 2024, 36, 2401369. 10.1002/adma.202401369 38822749

[2] M. Zhao , Y. Wang , L. Li , S. Liu , C. Wang , Y. Yuan , G. Yang , Y. Chen , J. Cheng , Y. Lu , J. Liu , Theranostics. 2021, 11, 1845. 33408785 10.7150/thno.50905 PMC7778599

[3] Y. Zhang , G. Chen , X. Zhuang , M. Guo , Oxid. Med. Cell Longev. 2021, 2021, 8807676. 35003521 10.1155/2021/8807676 PMC8736697

[4] a) Y. Lai , F. F. Gao , R. T. Ge , R. Liu , S. Ma , X. Liu , Cell Biol. Toxicol. 2024, 40, 72; 39162885 10.1007/s10565-024-09910-4 PMC11335907

[5] N. Bryan , H. Ahswin , N. Smart , Y. Bayon , S. Wohlert , J. A. Hunt , Eur. Cell Mater. 2012, 24, 249. 23007910 10.22203/ecm.v024a18

[6] J. A. Escobar , M. A. Rubio , E. A. Lissi , Free Radic. Biol. Med. 1996, 20, 285. 8720898 10.1016/0891-5849(95)02037-3

[7] B. Guo , F. Yang , L. Zhang , Q. Zhao , W. Wang , L. Yin , D. Chen , M. Wang , S. Han , H. Xiao , N. Xing , Adv. Mater. 2023, 35, 2212267. 10.1002/adma.202212267 36916030

[8] C. Paul , A. B. Donita , Mol. Cell. 2022, 82, 10.34995506

[9] P. Tsvetkov , S. Coy , B. Petrova , M. Dreishpoon , A. Verma , M. Abdusamad , J. Rossen , L. Joesch‐Cohen , R. Humeidi , R. D. Spangler , J. K. Eaton , E. Frenkel , M. Kocak , S. M. Corsello , S. Lutsenko , N. Kanarek , S. Santagata , T. R. Golub , Science. 2022, 375, 1254. 35298263 10.1126/science.abf0529 PMC9273333

[10] a) Z. Yang , N. Xuegang , D. Chengyu , L. Yuanxiang , F. Wenhua , Y. Lingjun , C. Junjie , Z. Jianhua , T. Yu , H. Wei , H. Wen , P. Yuanbo , W. Tiantian , C. Xiaoyuan , K. Dezhi , Adv. Sci. 2024, 11, 23;

[11] a) K. Raghu , L. Valerie , Science. 2020, 367, 6478;

[12] a) R. Isaac , F. C. G. Reis , W. Ying , J. M. Olefsky , Cell Metab. 2021, 33, 1744; 34496230 10.1016/j.cmet.2021.08.006 PMC8428804

[13] Q. Wei , J. Su , S. Meng , Y. Wang , K. Ma , B. Li , Z. Chu , Q. Huang , W. Hu , Z. Wang , L. Tian , X. Liu , T. Li , X. Fu , C. Zhang , Adv. Sci. 2024, 11, e2307761. 10.1002/advs.202307761 PMC10987139 38286650

[14] L. Alvarez‐Erviti , Y. Seow , H. Yin , C. Betts , S. Lakhal , M. J. Wood , Nat. Biotechnol. 2011, 29, 341. 21423189 10.1038/nbt.1807

[15] W. Yue , L. Xin , F. Xueyu , H. Xiaomin , Z. Shenrong , Z. Nan , M. Xiaowei , S. Qimanguli , Y. Mei , T. Wei , Z. Xingtao , H. Jinhai , Adv. Sci. 2024, 11, 32.

[16] a) S. Mura , J. Nicolas , P. Couvreur , Nat. Mater. 2013, 12, 991; 24150417 10.1038/nmat3776

[17] H. Wang , B. Wang , A. Zhang , F. Hassounah , Y. Seow , M. Wood , F. Ma , J. D. Klein , S. R. Price , X. H. Wang , Mol. Ther. 2019, 27, 571. 30711446 10.1016/j.ymthe.2019.01.008 PMC6403486

[18] a) X. Dong , Y. Lei , Z. Yu , T. Wang , Y. Liu , G. Han , X. Zhang , Y. Li , Y. Song , H. Xu , M. Du , H. Yin , X. Wang , H. Yan , Theranostics. 2021, 11, 5107; 33859737 10.7150/thno.54755 PMC8039955

[19] R. Mrowczynski , ACS Appl. Mater. Interfaces. 2018, 10, 7541. 28786657 10.1021/acsami.7b08392

[20] G. Lin , F. Zhu , N. M. Kanaan , R. Asano , N. Shirafuji , H. Sasaki , T. Yamaguchi , S. Enomoto , Y. Endo , A. Ueno , M. Ikawa , K. Hayashi , O. Yamamura , S. H. Yen , Y. Nakamoto , T. Hamano , Int. J. Mol. Sci. 2021, 22, 21. 10.3390/ijms222112063 PMC8584684 34769495

[21] X. Li , X. Peng , M. Zoulikha , G. F. Boafo , K. T. Magar , Y. Ju , W. He , Sig. Transduct. Target Ther. 2024, 9, 1. 10.1038/s41392-023-01668-1 PMC10758001 38161204

[22] B. Marta , K. Michael J , L. M. Tanguy , D. Hugh R W , S. Tobias , S. Loïc , F. Isabella C , E. Lyndon , P. Roberta , P. Guido , J. Am. Chem. Soc. 2020, 142, 46.

[23] K. Ma , J. Z. Cui , J. B. Ye , X. M. Hu , G. L. Ma , X. P. Yang , Food Chem. 2017, 230, 291. 28407913 10.1016/j.foodchem.2017.03.057

[24] T. Kekic , J. Lietard , Sci. Rep. 2022, 12, 14803. 36045146 10.1038/s41598-022-19069-9 PMC9428881

[25] F. Wang , L. Zeng , Y. Chi , S. Yao , Z. Zheng , S. Peng , X. Wang , K. Chen , Int. Immunopharmacol. 2024, 139, 112679. 39013217 10.1016/j.intimp.2024.112679

[26] X. Gao , N. Ran , X. Dong , B. Zuo , R. Yang , Q. Zhou , H. M. Moulton , Y. Seow , H. Yin , Sci. Transl. Med. 2018, 10, 444. 10.1126/scitranslmed.aat0195 29875202

[27] Z. Chu , Q. Huang , K. Ma , X. Liu , W. Zhang , S. Cui , Q. Wei , H. Gao , W. Hu , Z. Wang , S. Meng , L. Tian , H. Li , X. Fu , C. Zhang , Bioact. Mater. 2023, 27, 257. 37122894 10.1016/j.bioactmat.2023.04.007 PMC10133407

[28] Y. Zhujun , L. Xuejian , G. Xueqi , W. Mengying , W. Chunbao , Y. Guodong , Z. Yimin , Z. Zhuoli , W. Zhongshan , J. Nanobiotechnol. 2023, 21, 119.

[29] P. Chaoyu , W. Yong , X. Honglin , H. Jiangtao , S. Qiyuan , Y. Yuan , C. Lu , J. Ke , Y. Hanfeng , L. Yuling , J. Nanobiotechnol. 2024, 22, 1.

[30] Z. Chen , Y. Zou , H. Sun , Y. He , K. Ye , Y. Li , L. Qiu , Y. Mai , X. Chen , Z. Mao , C. Yi , W. Wang , Adv. Mater. 2024, 36, 45. 10.1002/adma.202412253 39295480

[31] S. Lianhui , Z. Yuan , Y. Boyu , S. Sijun , Z. Pengshan , L. Zai , F. Tingting , C. Zelin , Z. Ting , L. Yuming , Q. Zhengjun , F. Guangjian , H. Chen , Nat. Commun. 2023, 14, 1155.36859400

[32] M. Y. Hoover , T. H. Ambrosi , H. M. Steininger , L. S. Koepke , Y. Wang , L. Zhao , M. P. Murphy , A. A. Alam , E. J. Arouge , M. G. K. Butler , E. Takematsu , S. P. Stavitsky , S. Hu , D. Sahoo , R. Sinha , M. Morri , N. Neff , J. Bishop , M. Gardner , S. Goodman , M. Longaker , C. K. F. Chan , Nat. Protoc. 2023, 18, 2256. 37316563 10.1038/s41596-023-00836-5 PMC10495180

[33] W. Tao , Z. Jiafeng , P. Qi , Z. Tianhua , P. Yuan , L. Xiangrui , Sci. Adv. 2022, 8, 37.

[34] M. Wenjuan , Z. Xiaoxuan , L. Yuxiao , F. Lu , G. Jingjing , L. Weilin , Z. Yuanjin , S. Lingyun , Adv. Sci. 2022, 9, 13.

[35] X. Xuejiao , C. Jing , J. Tao , Y. Chengqi , K. Yu , Z. Maojie , X. Kaituo , G. Jiahe , J. Guoyong , W. Cheng , XiangXu, Y. X. , C. Zhenbing , Drug Deliv. Trans. Res. 2023, 13, 9.

[36] X. Ao , J. Xinran , H. Yongyan , L. Hu , J. Yanli , Y. Dedong , W. Yuqiong , S. Hong , W. Han , L. Long , C. Tianrui , L. Feng , Y. Kuan , H. Zhaocun , S. Yanan , Z. Penghua , F. Yao , K. Shenshen , M. Wei , W. Yi , Y. Xinge , C. Lingqian , Adv. Mater. 2024, 36, 40.

