# Supplementary material for: Nanodrug‐Engineered Exosomes Achieve a Jointly Dual‐Pathway Inhibition on Cuproptosis
Source: Adv Sci (Weinh). 2024 Dec 5;12(4):2413408. doi: 10.1002/advs.202413408 (PMC11775538; doi:10.1002/advs.202413408)
Supplement: Supplementary file 1 — Supporting Information [file ADVS-12-2413408-s001.docx]

Supporting Information

**Nanodrug-engineered Exosomes Achieve a Jointly Dual-pathway Inhibition on** **Cuproptosis**

*Hanxiao Sun ^a^ , Yang Zou ^b^, Zhengtai Chen ^a^, Yan He ^a^, Kai Ye ^a^, Huan Liu ^a^, Lihong Qiu ^a^, Yufan Zhang ^a^ , Yuexue Mai ^a^, Xinghong Chen ^a^ , Zhengwei Mao ^c^, Wei Wang ^a,b*^ , Chenggang Yi ^a*^*

Hanxiao Sun

a. The Second Affiliated Hospital of Zhejiang University College of Medicine, Hangzhou, 310000, China

Yang Zou

b. College of Chemical and Biological Engineering, Zhejiang University, Hangzhou Zhejiang 310027, China

Zhengtai Chen, Yan He, Kai Ye, Huan Liu, Lihong Qiu, Yufan Zhang, Yuexue Mai, Xinghong Chen, Chenggang Yi

a. The Second Affiliated Hospital of Zhejiang University College of Medicine, Hangzhou, 310000, China

Zhengwei Mao

c. MOE Key Laboratory of Macromolecular Synthesis and Functionalization, Department of Polymer Science and Engineering, Zhejiang University, Hangzhou, Zhejiang 310027, China.

Wei Wang

a. The Second Affiliated Hospital of Zhejiang University College of Medicine, Hangzhou, 310000, China

b. College of Chemical and Biological Engineering, Zhejiang University, Hangzhou Zhejiang 310027, China

E-mail: Corresponding author Wei Wang (wwgfz@zju.edu.cn); Chenggang Yi ([yichg@zju.edu.cn](mailto:yichg@zju.edu.cn)).

**Keywords:** Wound healing, cuproptosis, FDX1, engineered exosomes

**Supplementary Figures**

**
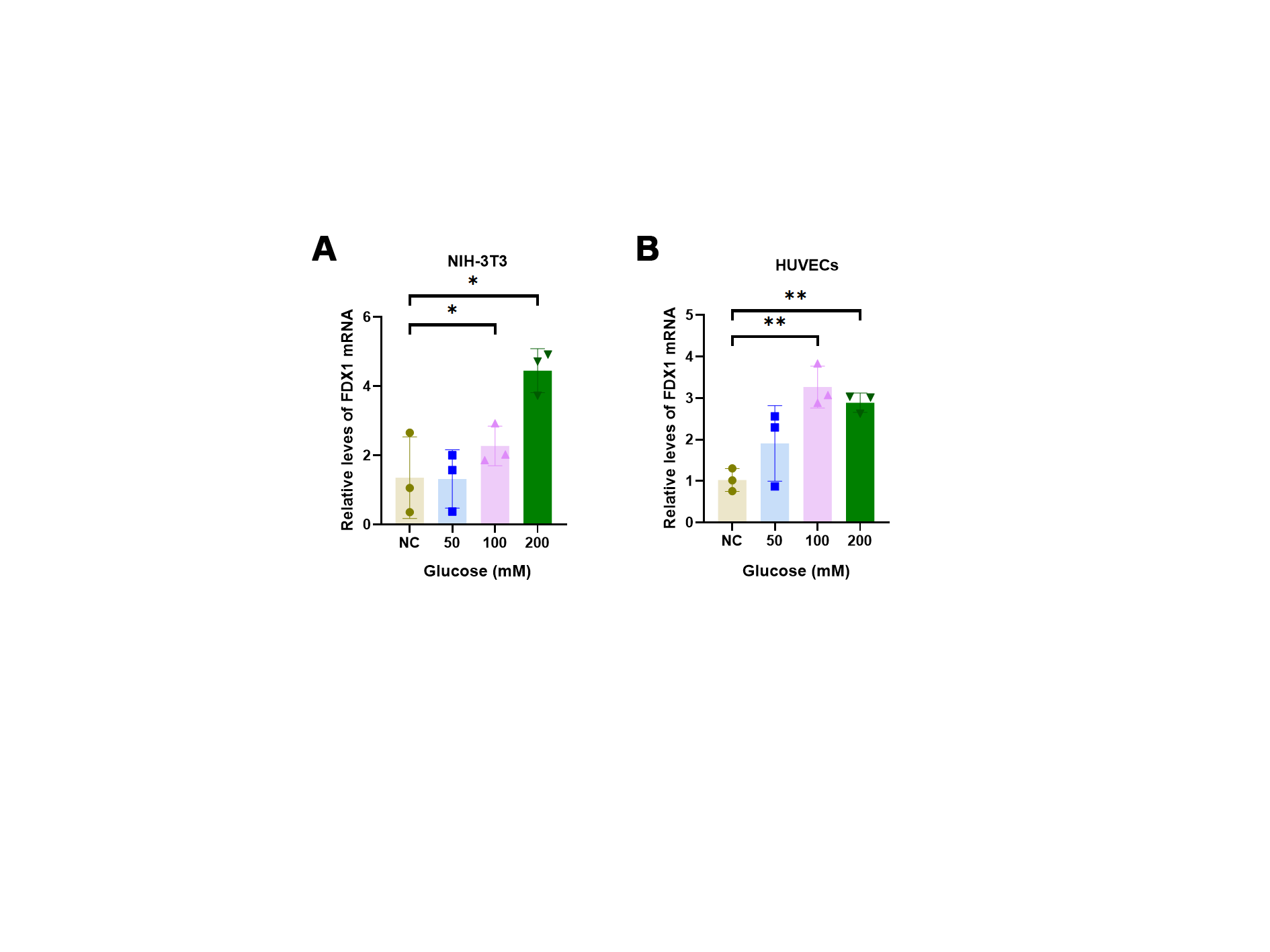
**

**Fig. R4. Expression levels of FDX1 in NIH-3T3 (A) and HUVECs (B).**

**
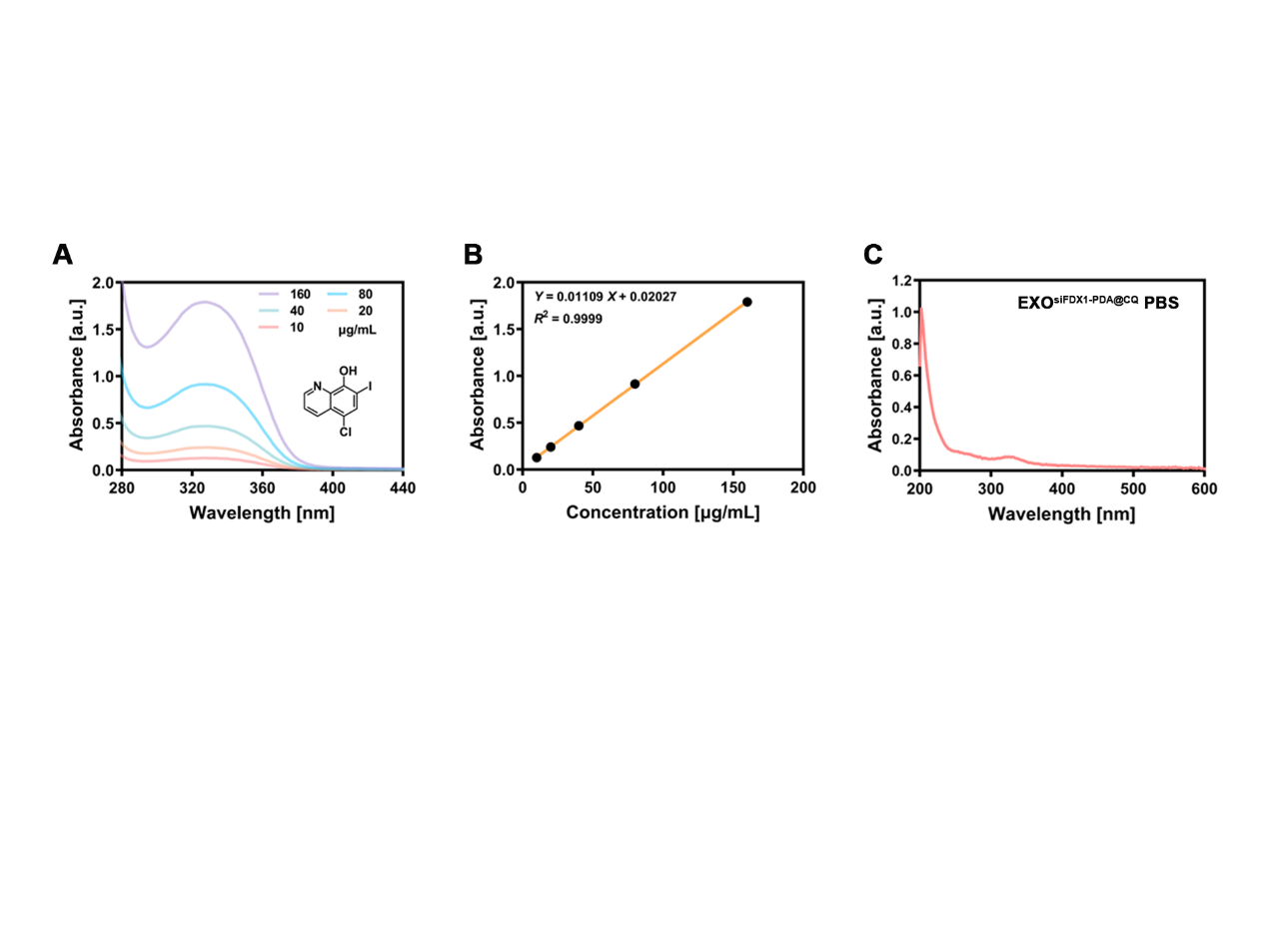
**

**Fig. S2.** **CQ content carried by EXO^siFDX1-PDA@CQ^.** (A) UV-Vis spectra of CQ. (B) Standard curve of CQ obtained by linear fitting. (C) UV-Vis spectrum of EXO^siFDX1-PDA@CQ^.


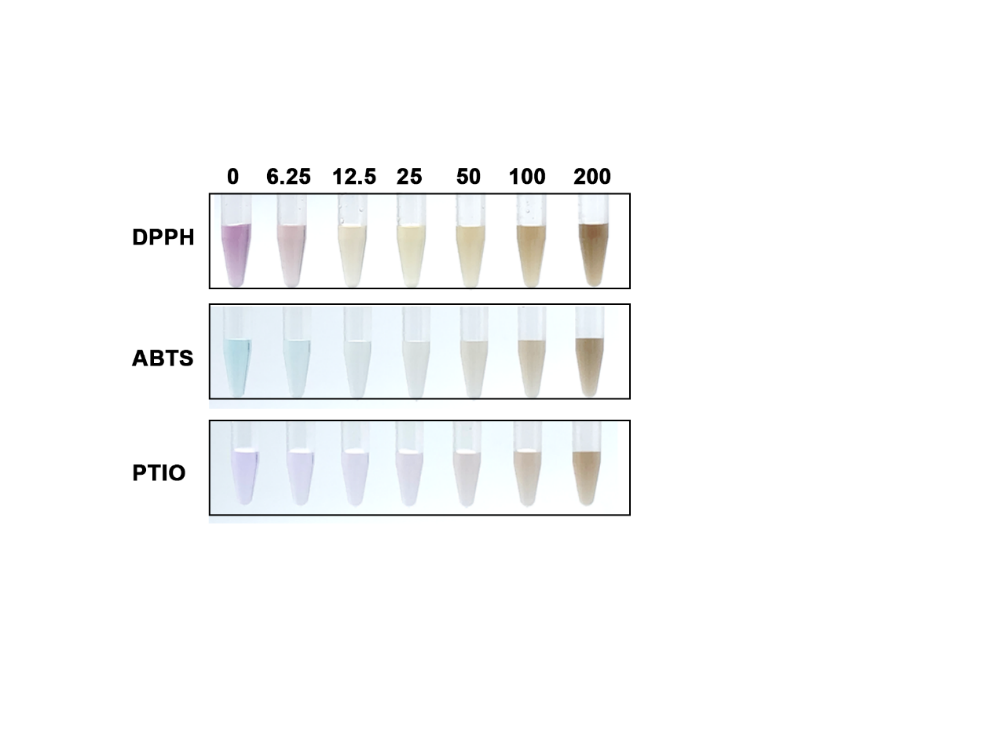


**Fig. S3.** **Representative images of various radical solutions after treatment with various concentrations of PDA@CQ NPs.**

OSA was synthesized via the chemical oxidation of SA **(Fig. S4A)**, and the relevant proton signals of the aldehyde group and sugar ring were recorded by ^1^H NMR **(Fig. S4B)**. As illustrated in **Fig. S4C**, the stretching vibrations of -CHO- are presented at 1732 cm^−1^ after the oxidation reaction with NaIO_4_, demonstrating the successful oxidation of SA. The weight-average molecular weight of OSA is 3.76 × 10^4^, which was determined by GPC .


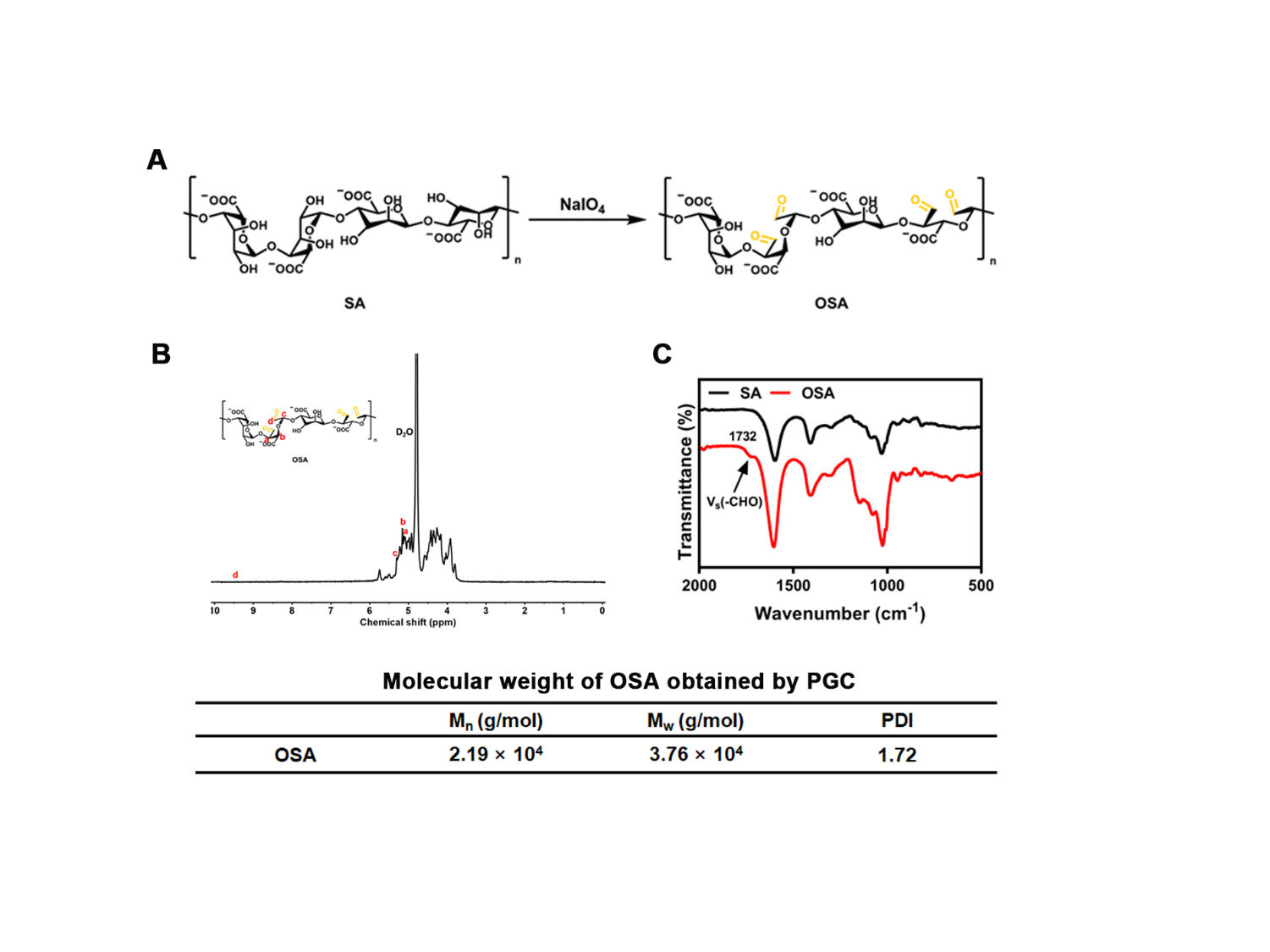


**Fig. S4. Synthesis and characterization of OSA.** (A) Synthesis route of OSA. (B) ^1^H NMR of OSA. (C) FTIR spectra of SA and OSA. Molecular weight of OSA obtained via GPC.

Parallelly, the chemical formation of PGA-g-ADH was evaluated via FTIR, ^1^H NMRspectroscopy, GPC, and zeta potential **(Fig. S5)**. PGA-g-ADH was prepared by amidation **(Fig. S5A)**, and a sharp signal centered at 1620 and 1270 cm^−1^, belonging to the stretching vibrations of -CO-NH- and -C-N- in PGA-g-ADH, respectively **(Fig. S5B)**. To confirm the chemical structure of PGA-g-ADH, we measured ^1^H NMR, and the peaks at 1.6 and 2.3 ppm are ascribed to the protons of the alkyl chains of ADH. The weight-average molecular weight of PGA-g-ADH is 2.41 × 10^5^, which was determined by GPC **(Fig. S5C)**. Additionally, the zeta potential of PGA-g-ADH is slightly improved than that of PGA, purporting the grafting of positive ADH on PGA backbones **(Fig. S5D)**.

**
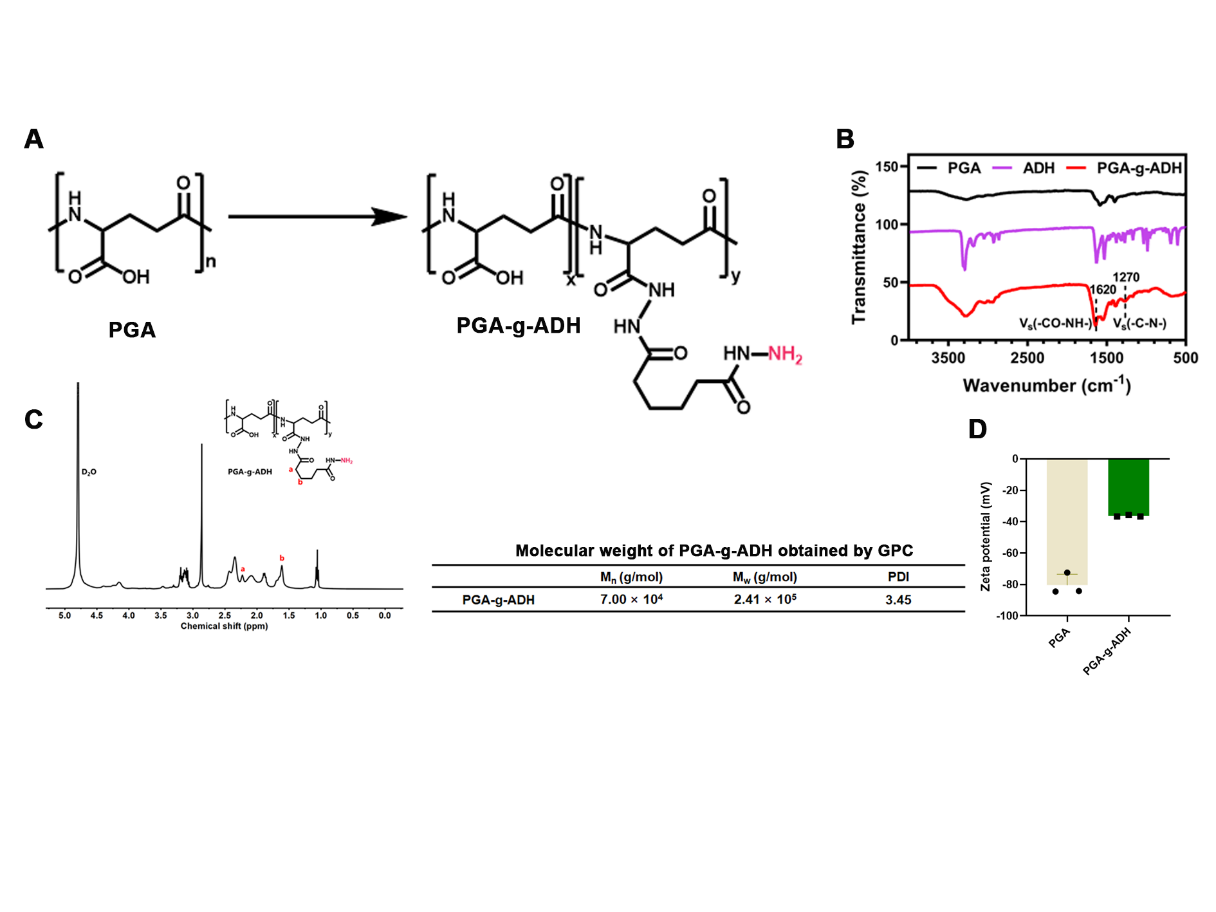
**

**Fig. S5. Synthesis and characterization of PGA-g-ADH.** (A) Synthesis route of PGA-g-ADH. (B) FTIR spectra of PGA, ADH, and PGA-g-ADH. (C) ^1^H NMR spectroscopy of PGA-g-ADH. Molecular weight of PGA-g-ADH obtained via GPC. (D) Zeta potential of PGA and PGA-g-ADH.


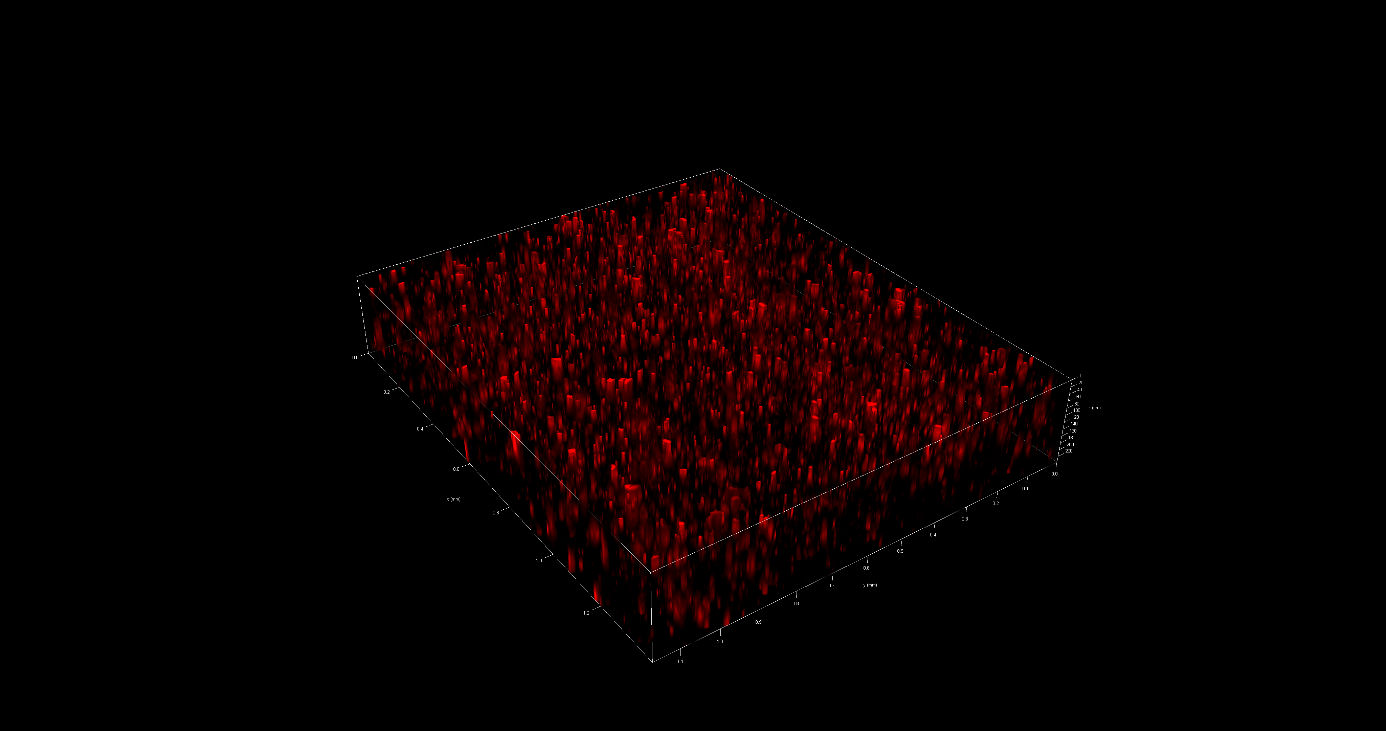


**Fig. S6. Distribution of EXO^siFDX1-PDA@CQ^ in hydrogel.** Confocal 3D scanning images of the hydrogel, which is embedded with EXO^siFDX1-PDA@CQ^ (red), show dimensions of 250 μm in height, 1.2 mm in width, and 1.4 mm in length.


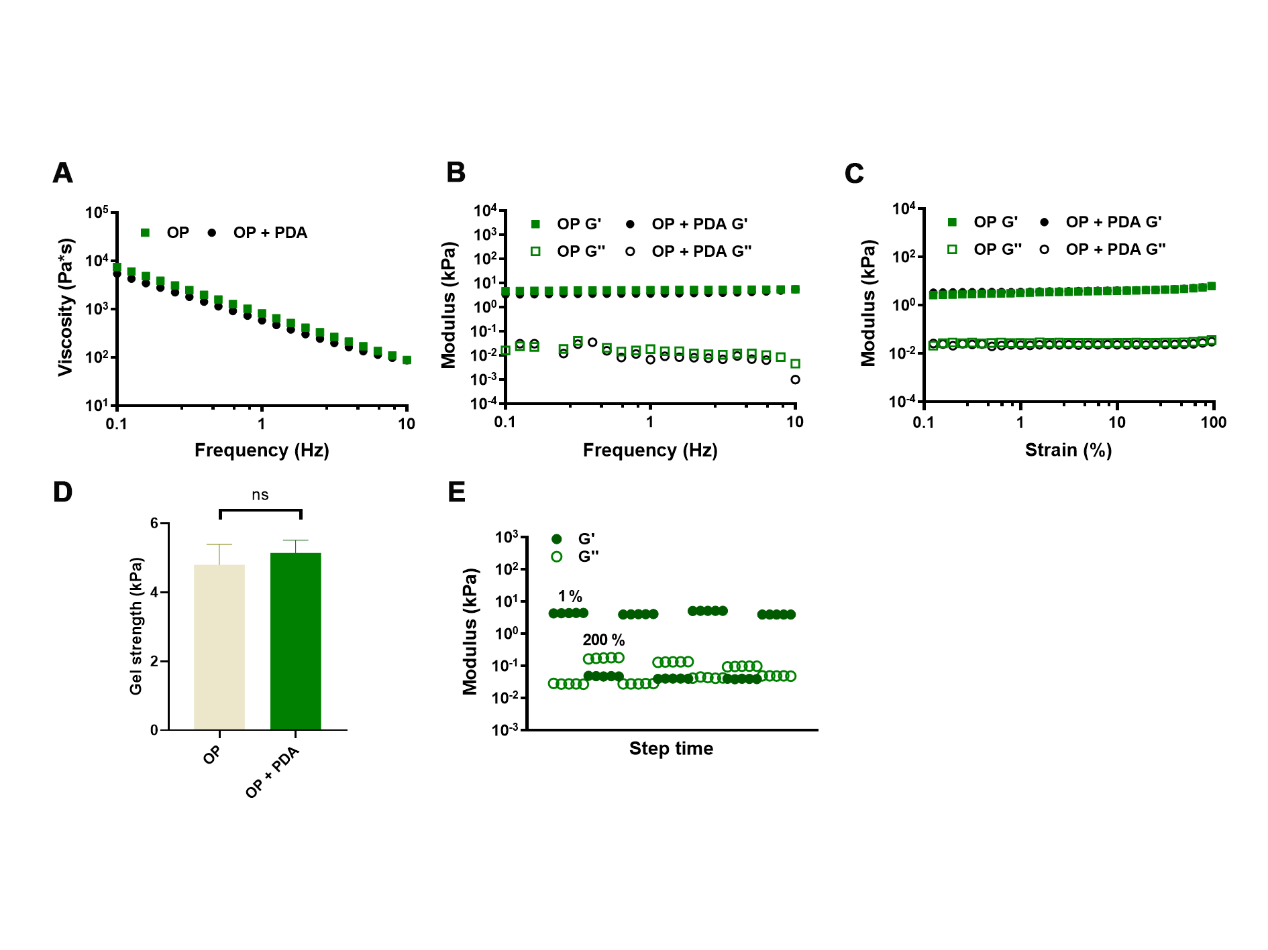


**Fig. S7. Rheological properties of hydrogel.** (A) Investigation of the shear-thinning properties exhibited by OP and OP + PDA hydrogel. (B) Evaluation of frequency-responsive rheological characteristics of OP and OP + PDA hydrogel. (C) Analysis of strain-responsive rheological behaviors of OP and OP + PDA hydrogel. (D) Gel strength of OP and OP + PDA hydrogel. (E) Variation in G’ and G’’ of the OP hydrogel under the alternate steep strain from small strain (𝛾 = 1%) to large strain (𝛾 = 200%).


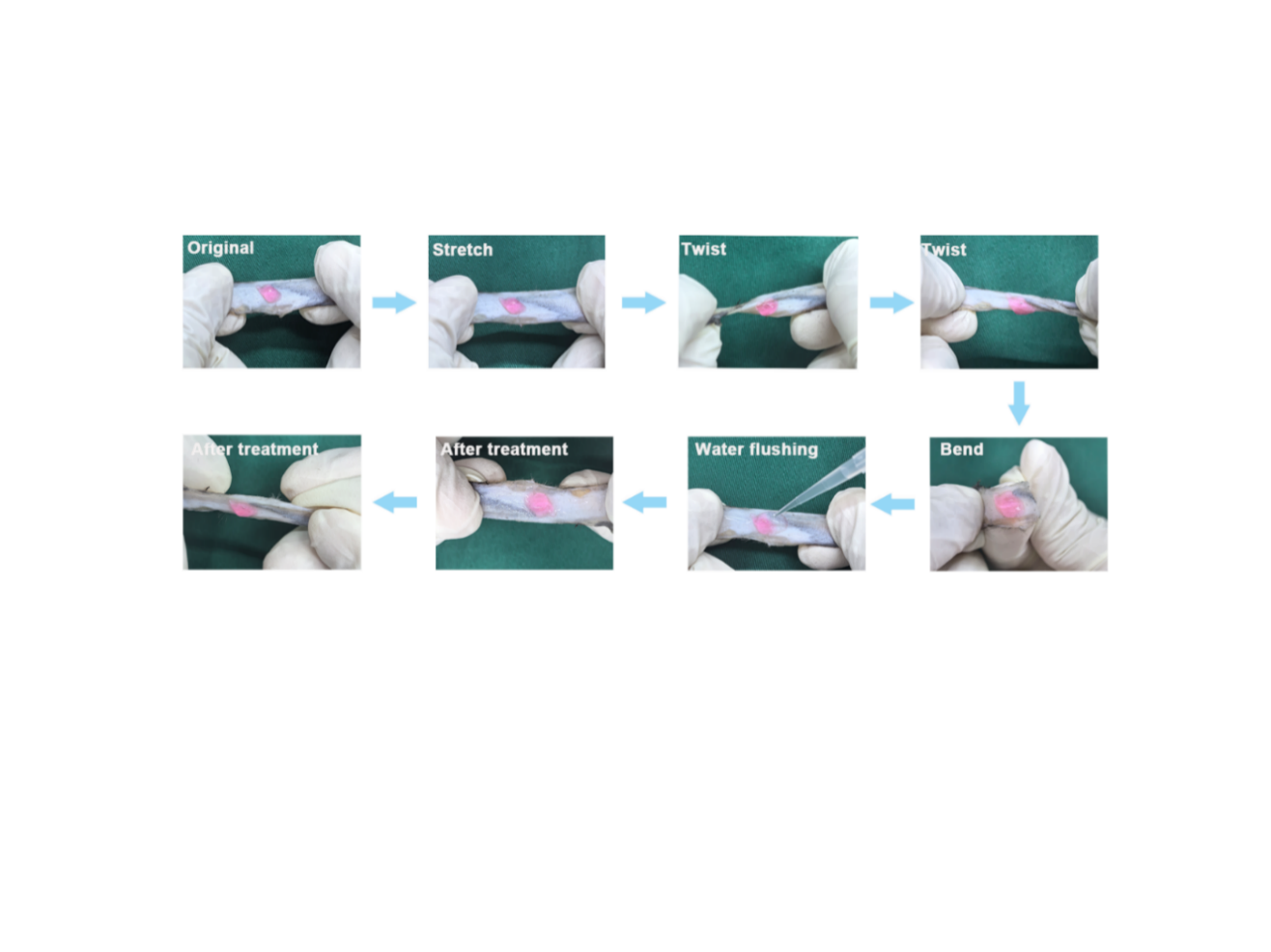


**Fig. S8. Adhesion of hydrogel to mouse skin.**


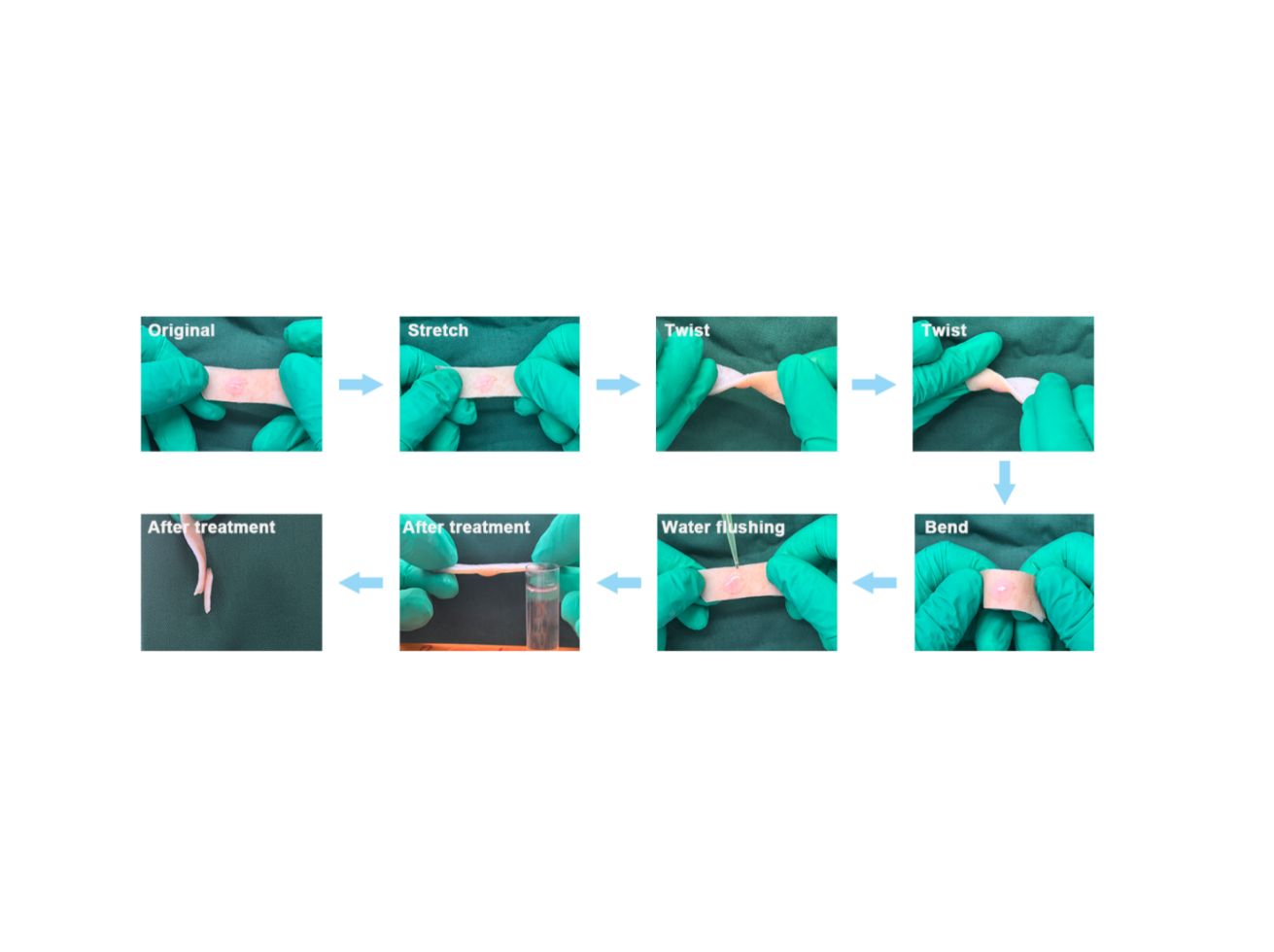


**Fig. S9. Adhesion of hydrogel to pig skin.**

Moreover, we investigated the anti-ROS performance of the resulting hydrogels through elimination measurements of DPPH and ABTS as model radicals **(Fig. S10)**. As demonstrated in **Fig. S10A** and **B**, the as-prepared pristine OP hydrogel alone (G-2) and gel with CP05 Pep (G-3) have restricted DPPH clearance. However, the addition of PDA@CQ NPs (G-4) turned the purple DPPH solution to yellow via a 30-min co-incubation, suggesting a remarkable DPPH-eliminating capability with the assistance of PDA@CQ NPs. A similar scenario of ABTS clearance was recorded, and all three experimental groups can transfer the color of the ABTS solution to colorless from light blue with a 30-min treatment, indicating a potent anti-ABTS property of our prepared OP hydrogels. In stark contrast to DPPH alone (G-1), the UV-vis absorption of DPPH/Gel dropped dramatically within 30 min **(Fig. S10C)**, and the average DPPH scavenging efficiency can be calculated as roughly 40% for G-2 and G-3, above 80% for G-4 **(Fig. S10D)**. Compared with control (G-1), the UV-vis absorption of the co-incubation group fell markedly in 30 min, as represented in **Fig. S10E**. Meanwhile, the average ABTS scavenging efficiency can be calculated as approximately 80% of all three groups (G-2-G-4, **Fig. S10F**). The collective outcomes indicated that our elaborately designed injectable OP hydrogels have macroscopic ROS-scavenging performance, and the synergy of PDA@CQ NPs with blank OP hydrogel enables the systematic dressings to function as antioxidant therapeutics in clinical practice.


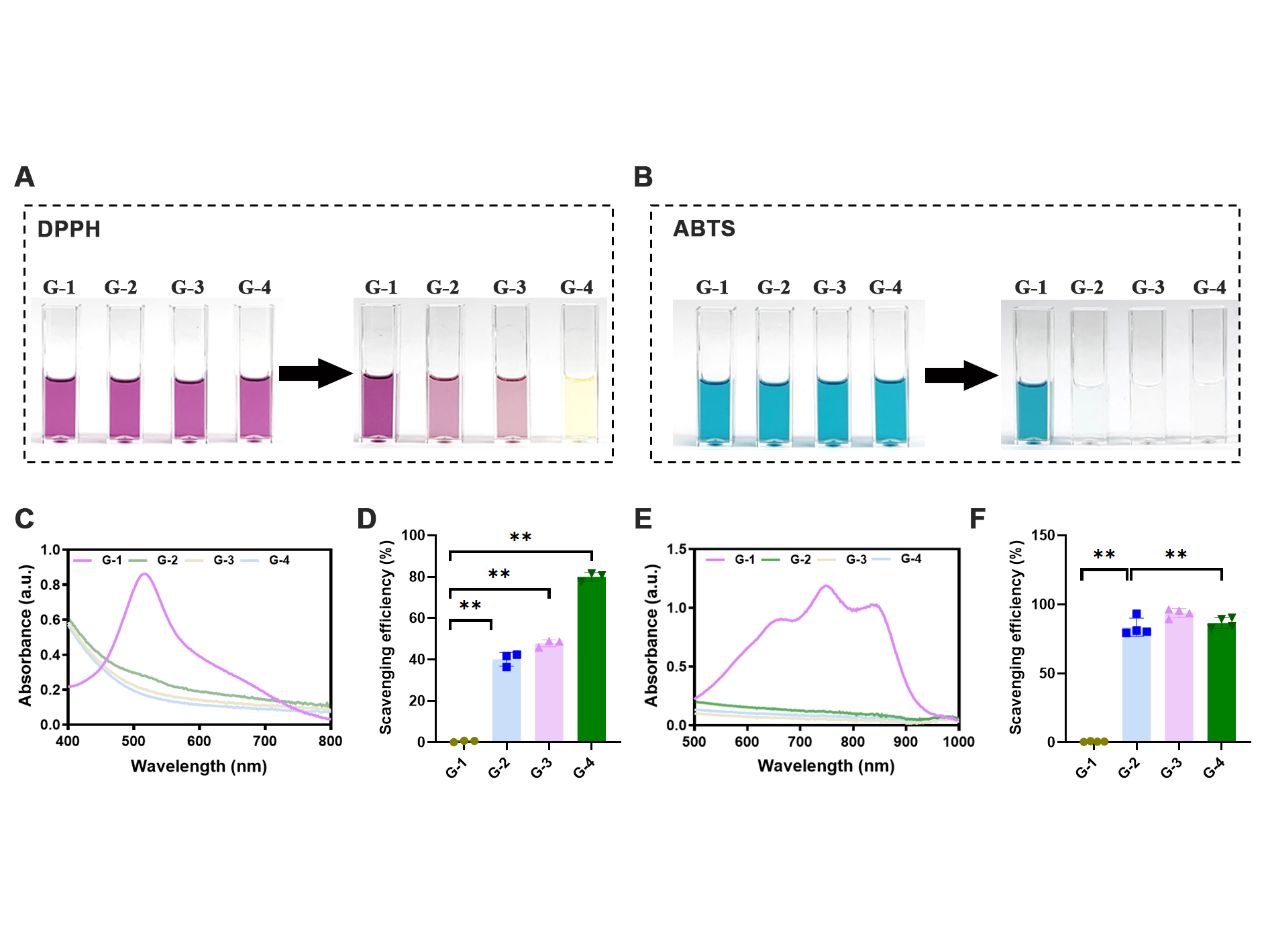


**Fig. S10. Analysis of ROS scavenging capabilities of hydrogel.** (A) Images of the initial DPPH solution and after 30 min co-culturing with hydrogel. G-1: control. G-2: OP hydrogel. G-3: OP + CP05 hydrogel. G-4: OP + CP05 + PDA hydrogel. (B) Images of the initial ABTS solution and after 30 min co-incubating with hydrogel (C) UV-vis absorption spectra of different DPPH solutions. (D) DPPH radical scavenging efficiency of hydrogel. (E) UV-vis absorption curves of various ABTS solutions. (F) ABTS radical scavenging efficiency of hydrogel.


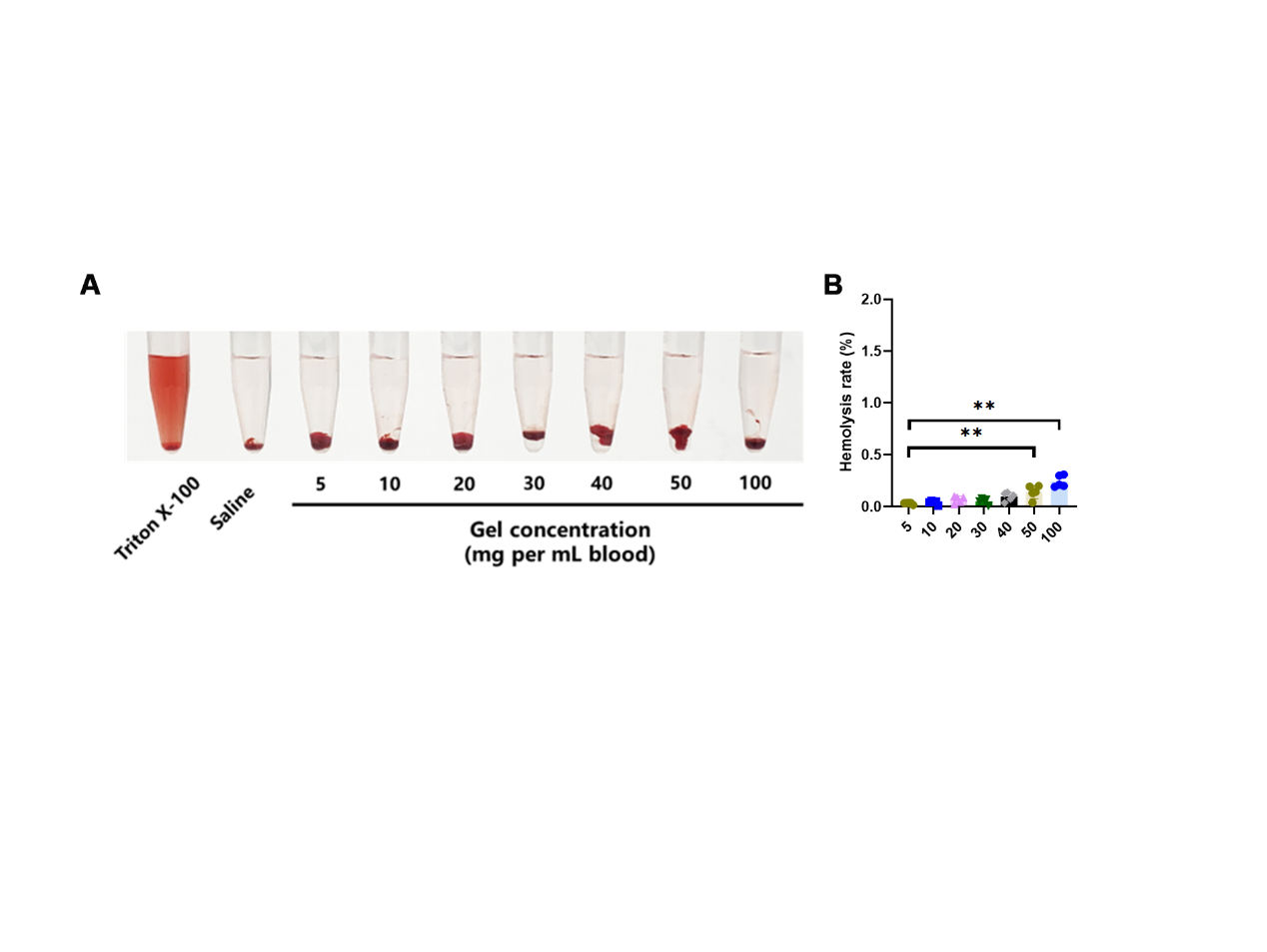


**Fig. S11. Characterization of the hemocompatibility of OP hydrogel.** (A) Image of hemolysis performance of the customized OP hydrogel. (B) Hemolysis rates of various customized OP hydrogel.


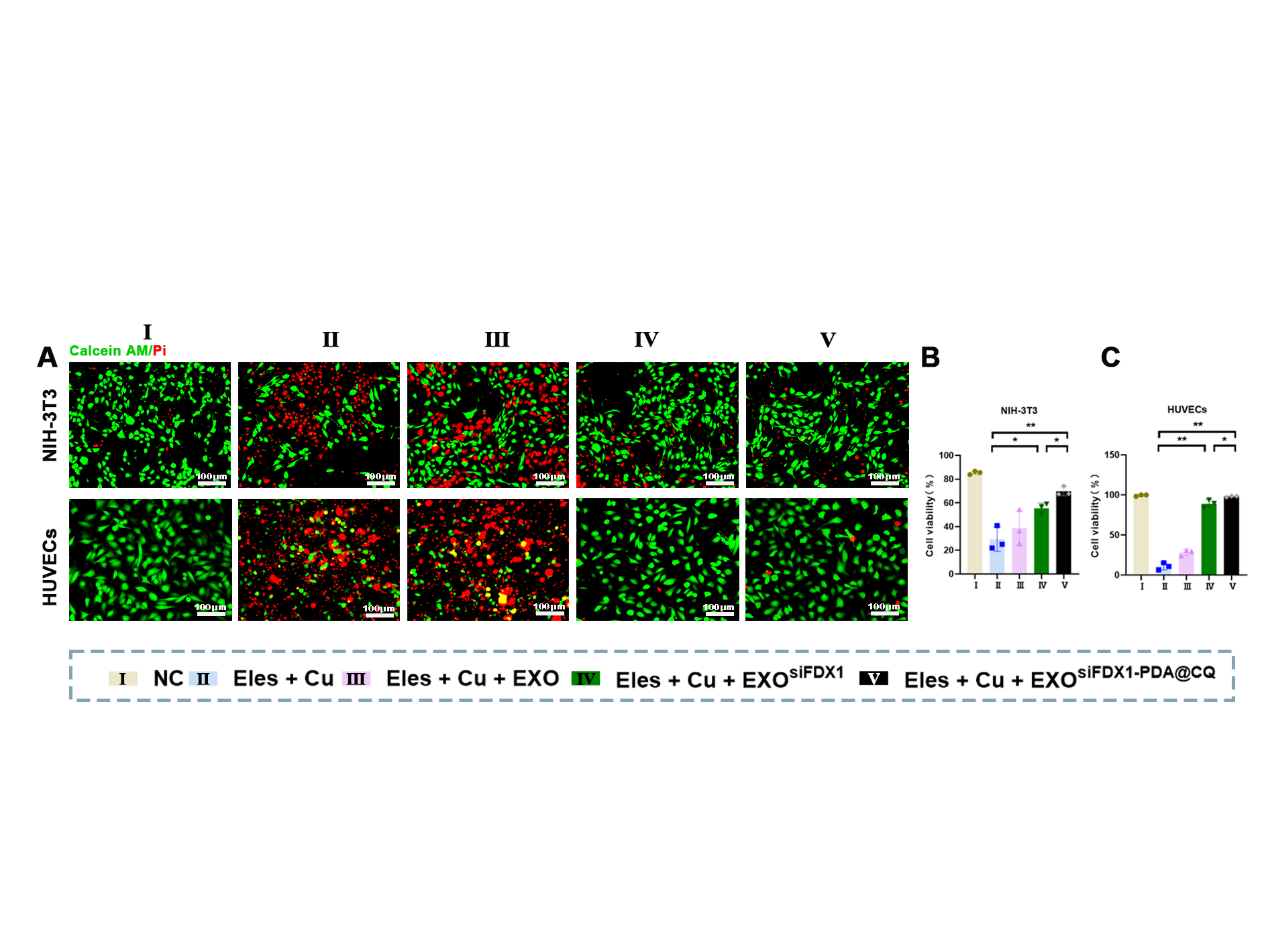


**Fig. S12. Cell viability staining detection of EXO^siFDX1-PDA@CQ^ inhibiting cell death.** (A) Fluorescence microscopy was used to detect the live/dead staining of NIH-3T3 and HUVECs. Scale bar = 100 µm. (B) Statistical analysis of live/dead results in NIH-3T3. (C) Statistical analysis of live/dead results in HUVECs.


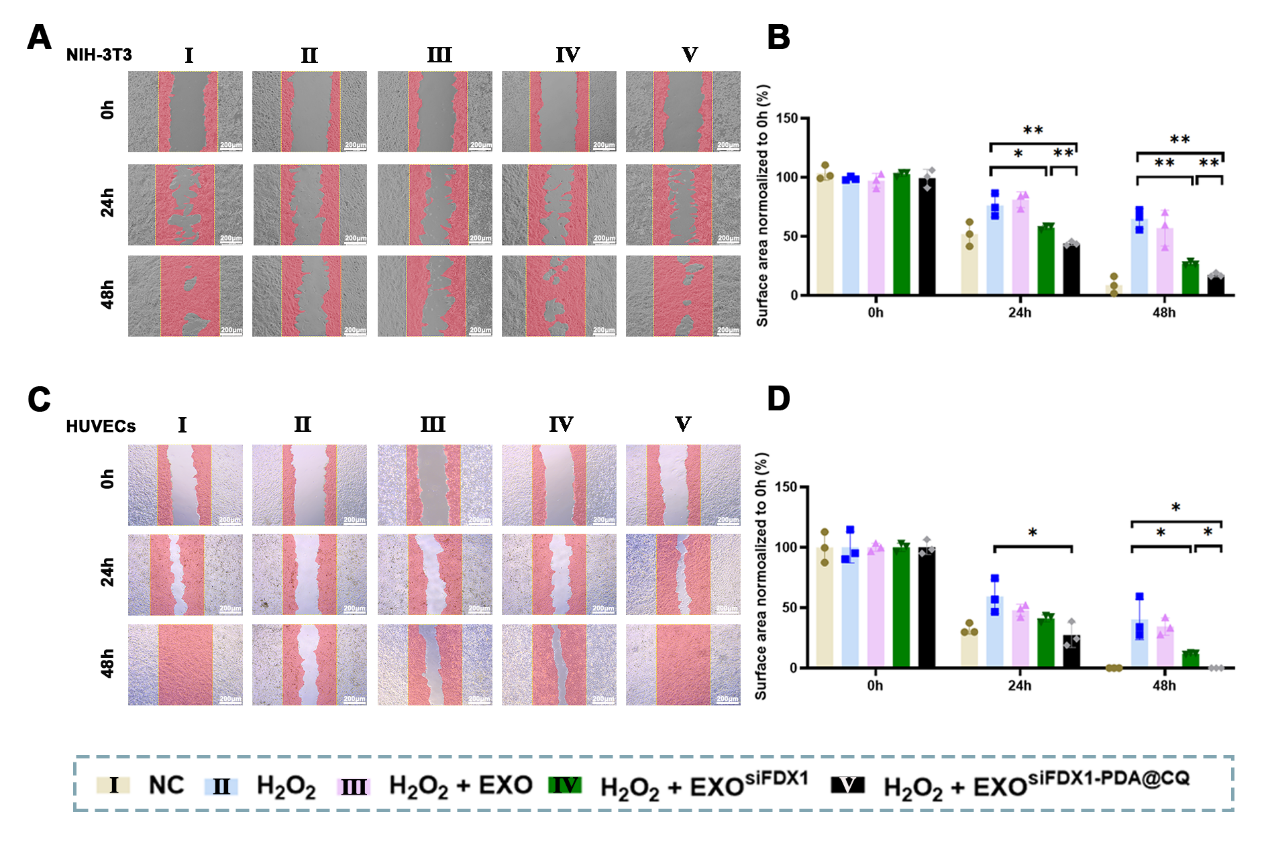


**Fig. S13.** **Scratch assay was** **conducted to evaluate the cell migration ability under H_2_O_2_ induced** **cuproptosis after corresponding intervention.** (A) NIH-3T3 migration was observed under light microscope. Scale bar =200 μm. (B) NIH-3T3 scratch results statistics. (C) HUVECs migration was observed under light microscope. Scale bar =200 μm. (D) HUVECs scratch statistics.


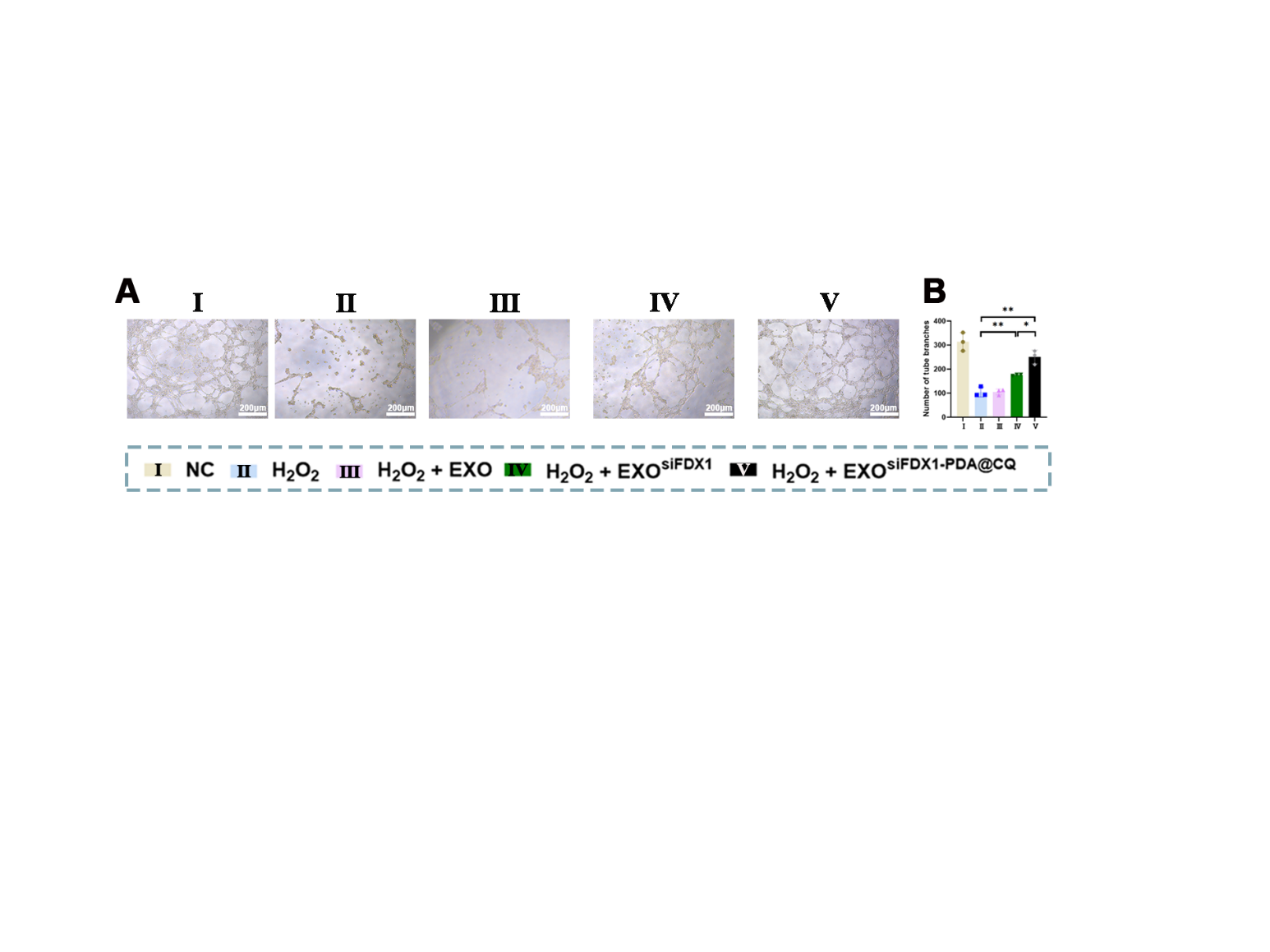


**Fig. S14. The tube forming test detected the tube forming ability of HUVECs ability under H_2_O_2_ induced** **cuproptosis.** (A) The tube formation of HUVECs was observed under light microscope. Scale bar =200 μm. (B) Statistical data on HUVECs tube formation.


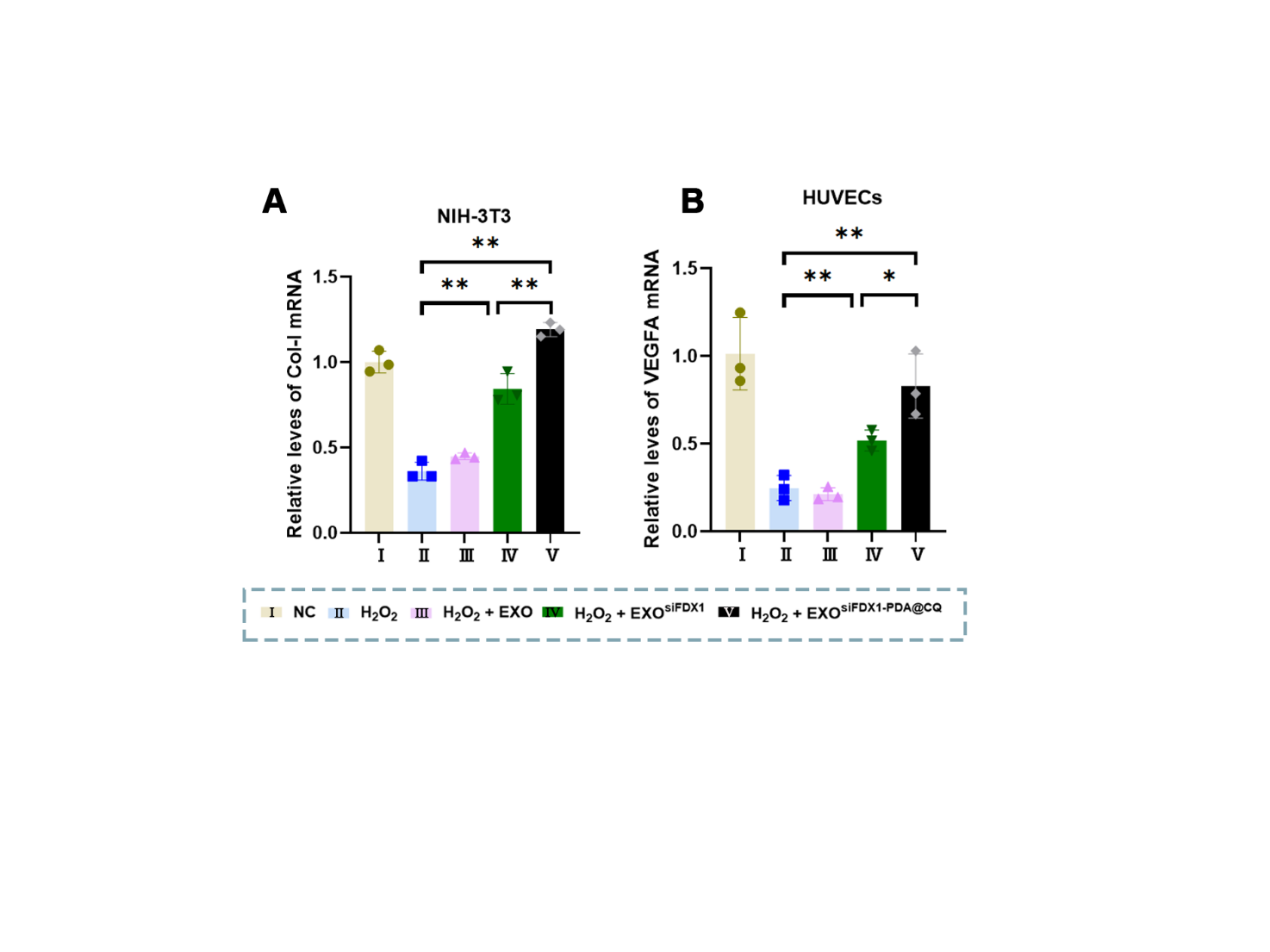


**Fig. S15.** **Gene expression levels of Col-Ⅰ and VEGFA in cells.** (A) The expression level of Col-Ⅰ in NIH-3T3 in each group. (B) The expression level of VEGFA in HUVECs in each group.


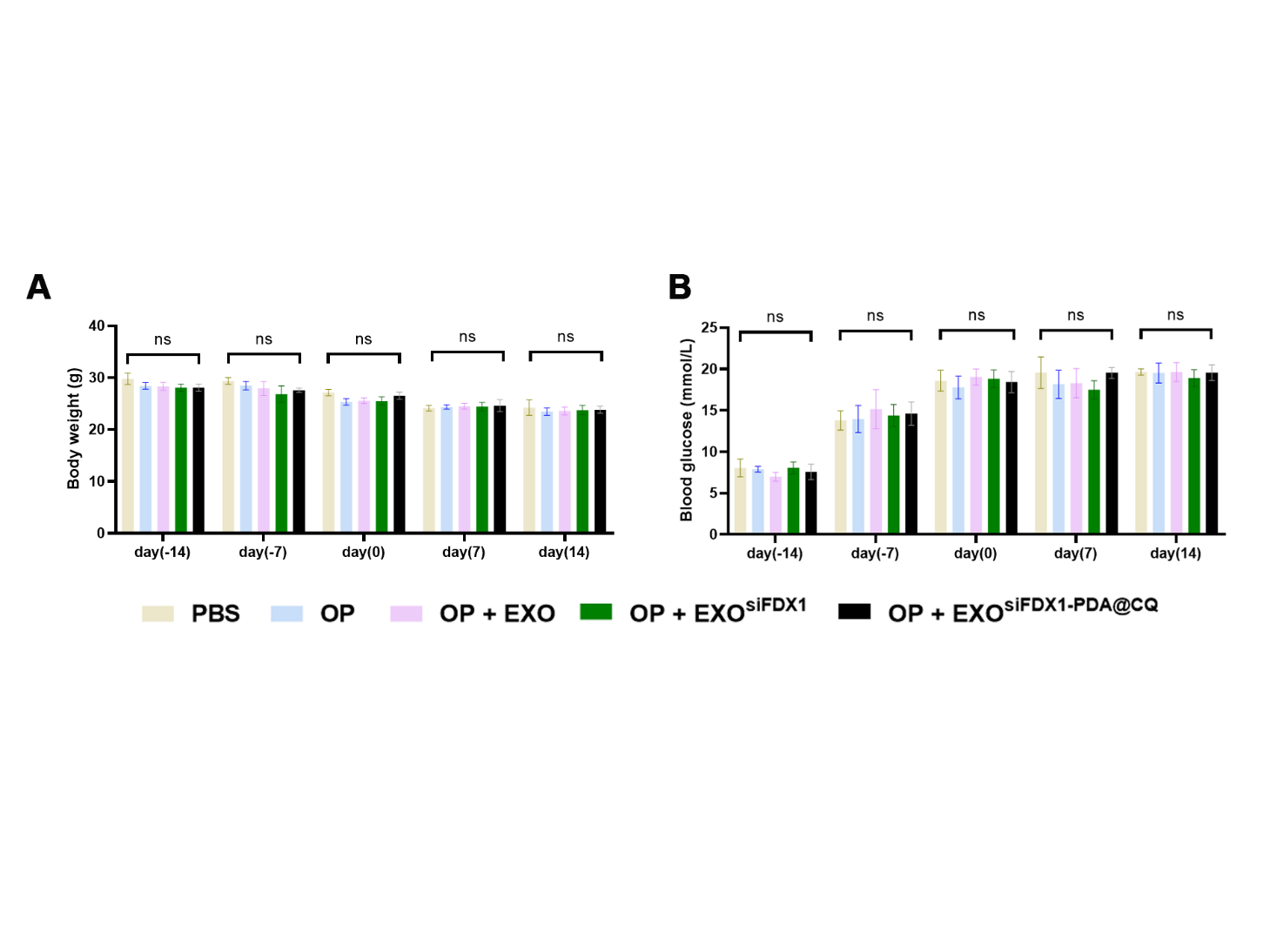


**Fig. S16.** **Body weight and blood glucose of mice in different time periods during the experiment.** (A) Changes in the body weight of mice at different time points. (B) Changes on blood glucose levels in mice at different time points.


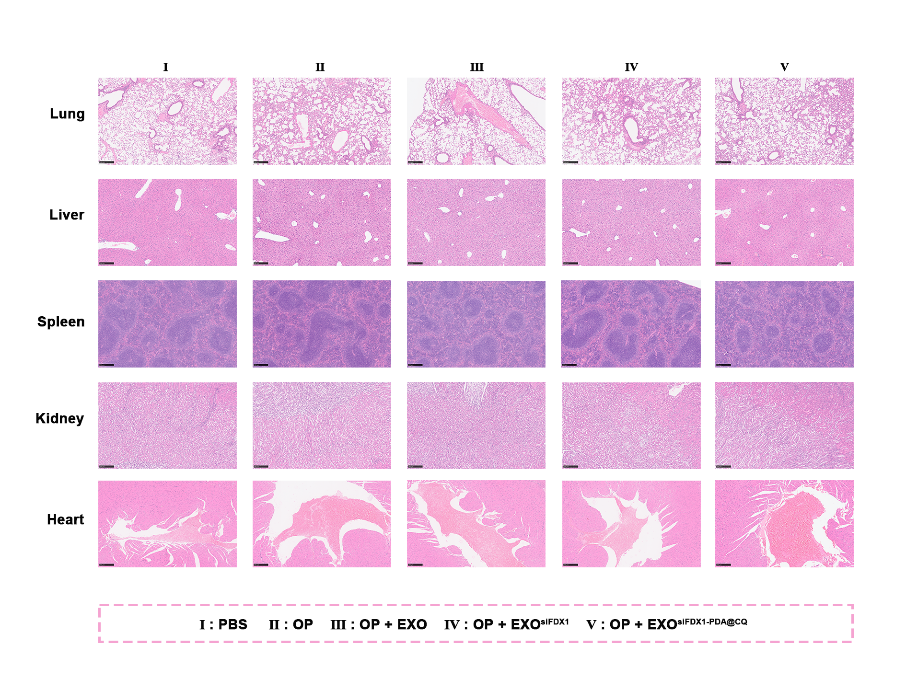


**Fig. S17. Biological safety assessment in mice.** H&E staining of heart, liver, spleen, lungs, and kidneys of mice on day 14. Scale bar = 250 µm.

**Table S1.** The siFDX1 sequence used in this study.

| **Gene** | **Sense (5’-3’)** | **Antisense (3’-5’)** | **Species** |
| --- | --- | --- | --- |
| FDX1-1 | GUGAUUGAGAACAACUUAGTT | CUAAGUUGUUCUCAAUCACTT | Mouse |
| FDX1-2 | GCUCUACUUGUCAUCUUAUTT | AUAAGAUGACAAGUAGAGCTT | Mouse |
| FDX1 | CGUGAUGGUAAGACGUUAATT | UUAACGUCUUACCAUCACGTT | Pig |

**Table S1. The utilized siFDX1 sequence**

**Table S2.** Primers employed in this study.

| **Gene** | **Forward** | **Reverse** | **Species** |
| --- | --- | --- | --- |
| FDX1  GAPDH | GAACTGCCATCTCTCCGTGG  GCATCTTCTTGTGCAGTGCC | CTCGCCATCTCGGTTCTTGA  TACGGCCAAATCCGTTCACA | Mouse  Mouse |
| FDX1  GAPDH | TTCAACCTGTCACCTCATCTTTG  GGAGCGAGATCCCTCCAAAAT | TGCCAGATCGAGCATGTCATT  GGCTGTTGTCATACTTCTCATGG | Human  Human |
| FDX1  GAPDH  Col-I  VEGFA | GGACCTTGGCTTGCTCTACCTG  GGCTGTGGGCAAGGTCATCC  GCTCCTCTTAGGGGCCACT  AGGGCAGAATCATCACGAAGT | CATTCTCCTCATCGGTGATTGCTTC  TCTCCAGGCGGCAGGTCAG  ATTGGGGACCCTTAGGCCAT  AGGGTCTCGATTGGATGGCA | Pig  Pig  Mouse  Human |

**Table S2. Gene Primers for Real-time Ploymerase Chain Reaction**
